# Supplementary material for: Illness prevalence and symptoms in youth floorball players: a one-season prospective cohort study involving 471 players
Source: BMJ Open. 2021 Dec 13;11(12):e051902. doi: 10.1136/bmjopen-2021-051902 (PMC8671975; doi:10.1136/bmjopen-2021-051902)
Supplement: Author's manuscript [file bmjopen-2021-051902.draft_revisions.pdf]

BMJ Open is committed to open peer review. As part of this commitment we make the peer review history of every article we publish publicly available.

When an article is published we post the peer reviewers' comments and the authors' responses online. We also post the versions of the paper that were used during peer review. These are the versions that the peer review comments apply to.

The versions of the paper that follow are the versions that were submitted during the peer review process. They are not the versions of record or the final published versions. They should not be cited or distributed as the published version of this manuscript.

BMJ Open is an open access journal and the full, final, typeset and author-corrected version of record of the manuscript is available on our site with no access controls, subscription charges or pay-per-view fees (http://bmjopen.bmj.com).

If you have any questions on BMJ Open's open peer review process please email info.bmjopen@bmj.com

BMJ Open

Prevalence, seasonal variation and nature of illness in youth floorball players – a one-season prospective cohort study involving 471 players.

Journal:	*BMJ Open*
Manuscript ID	bmjopen-2021-051902
Article Type:	Original research
Date Submitted by the Author:	01-Apr-2021
Complete List of Authors:	Panagodage Perera, Nirmala; Linkopings universitet<br>Waldén, Markus; Linkopings universitet<br>Lindblom, Hanna ; Linkopings universitet<br>Åkerlund, Ida; Linkopings universitet<br>Sonesson, Sofi; Linkopings universitet<br>Hagglund, Martin; Linkopings universitet
Keywords:	EPIDEMIOLOGY, SPORTS MEDICINE, PUBLIC HEALTH

SCHOLARONE™
Manuscripts

BMJ

I, the Submitting Author has the right to grant and does grant on behalf of all authors of the Work (as defined in the below author licence), an exclusive licence and/or a non-exclusive licence for contributions from authors who are: i) UK Crown employees; ii) where BMJ has agreed a CC-BY licence shall apply, and/or iii) in accordance with the terms applicable for US Federal Government officers or employees acting as part of their official duties; on a worldwide, perpetual, irrevocable, royalty-free basis to BMJ Publishing Group Ltd ("BMJ") its licensees and where the relevant Journal is co-owned by BMJ to the co-owners of the Journal, to publish the Work in this journal and any other BMJ products and to exploit all rights, as set out in our licence.

The Submitting Author accepts and understands that any supply made under these terms is made by BMJ to the Submitting Author unless you are acting as an employee on behalf of your employer or a postgraduate student of an affiliated institution which is paying any applicable article publishing charge ("APC") for Open Access articles. Where the Submitting Author wishes to make the Work available on an Open Access basis (and intends to pay the relevant APC), the terms of reuse of such Open Access shall be governed by a Creative Commons licence – details of these licences and which Creative Commons licence will apply to this Work are set out in our licence referred to above.

Other than as permitted in any relevant BMJ Author's Self Archiving Policies, I confirm this Work has not been accepted for publication elsewhere, is not being considered for publication elsewhere and does not duplicate material already published. I confirm all authors consent to publication of this Work and authorise the granting of this licence.

For peer review only

Prevalence, seasonal variation and nature of illness in youth floorball players – a one-season prospective cohort study involving 471 players.

Perera N KP, [1,2,3] Waldén M,[2,4,5] Lindblom H, [1,2] Åkerlund I, [1,2] Sonesson S, [1,2] Hägglund M[1,2]

[1]Unit of Physiotherapy, Department of Health, Medicine and Caring Sciences (HMV), Linköping University, Linköping, Sweden

[2] Sport Without Injury ProgrammE (SWIPE), Department of Health, Medicine and Caring Sciences (HMV), Linköping University, Linköping, Sweden

[3] Sport Medicine, The Australian Institute of Sport, Burce, ACT, Australia

[4] Unit of Community Medicine, Department of Health, Medicine and Caring Sciences (HMV), Linköping University, Linköping, Sweden

[5] Department of Orthopaedics, Hässleholm-Kristianstad Hospitals, Hässleholm, Sweden

Nirmala Perera perera.nk@outlook.com

Markus Waldén markus.walden@telia.com

Hanna Lindblom hanna.lindblom@liu.se

Ida Åkerlund ida.akerlund@liu.se

Sofi Sonesson sofi.sonesson@liu.se

Martin Hägglund (corresponding author) martin.hagglund@liu.se Unit of Physiotherapy, Department of Health, Medicine and Caring Sciences (HMV), Linköping University, 581 83 Linköping, Sweden. Tel +46 733347704

Keywords: Sport, Illness, Youth, Epidemiology

Word count: 2418

For peer review only

Abstract

Objective: To describe weekly illness prevalence and illness symptoms by sex in youth floorball players during one season.

Design: Prospective cohort study

Setting: Players who were registered to play community level floorball during the 2017–2018 season (26 weeks) from two provinces of southern Sweden.

Participants: 471 youth players aged 12–17 years. Mean age for 329 male players 13.3 years (SD 1.0) and 142 female players 13.7 years (SD 1.5).

Primary and secondary outcome measures: Weekly self-reported illness prevalence and illness symptoms, arranged according to the 2020 International Olympic Committee consensus recommendations.

Results: 61% of youth floorball players reported at least one illness week during the season, with an average weekly illness prevalence of 12% (95% CI 10.8-12.3%). The prevalence was slightly higher among females (13%, 95% CI 11.6-14.3%) than males (11%, 95% CI 9.9-11.7%), prevalence rate ratio 1.20 (95% CI 1.05-1.37, p=0.009). In total, 49% (53% male, 43% female) of illness reports indicated that the player could not participate in floorball (time loss). Upper/lower respiratory symptoms such as fever (30%), sore throat (16%) and cough (14%) were most common. More female players (16% vs 3% males, p<0.001) reported difficulty of breathing/tight airways. Illness prevalence was highest in the peak winter months (late January/February) reaching 15% - 18% in the period.

Conclusions: Our novel findings of the prevalence and pattern of illness in youth floorball may help direct prevention strategies. Flu-like symptoms dominated. Athletes, coaches, parents and support personnel need to be educated about risk mitigation in viral infections.

Trial registration: Clinical Trials registration NCT03309904

For peer review only

Article Summary – Strengths and limitations of this study

- The first study to evaluate self-reported prevalence, seasonal variations, and illness symptom clusters by sex according to the 2020 International Olympic Committee methods for recording and reporting epidemiological data consensus recommendations.
- Illness data from a large sample of male and female recreational youth floorball players over a 26-week period.
- Self-reported illness data may be subject to recall and interpretation bias, and the actual diagnosis was not available.
- Injury was the primary outcome of interest in the intervention study; players who reported an injury in the weekly survey could not report a concurrent illness in the same week, and illness data thus represent minimum illness prevalence rates in the study cohort.

Funding statement

The Sport Without Injury ProgrammE is funded by the Swedish Research Council (2015-02414 and 2018-03135) and the Swedish Research Council for Sport Science (P2018-0167).

Competing interests statement

None declared.

For peer review only - http://bmjopen.bmj.com/site/about/guidelines.xhtml

Introduction

Acute illness presents a significant health burden to athletes, interfere with their training, reduce sport performance, cause time loss from competitions [1-3] and may increase the risk of subsequent injuries.[4] Strenuous exercise during acute infection can increase the risk of serious medical complications, including sudden death.[2 5-7] Performance impairments from upper respiratory tract illness may also last for 2–4 days after complete recovery.[2] Infective illness can reduce muscle strength and impair motor coordination,[7] decrease aerobic and endurance capacity[5] and alter metabolic function[7] during and/or after the infection.

In elite youth sports, illness prevalence is higher in athletes who participate in endurance sports (23%) than those who participate in technical (10%) and team (8%) sports.[8] In elite adult sports, illness is more common in female athletes and those who participate in outdoor winter sports;[9] however, this relationship is unclear in elite youth sports.[10-13] The incidence of illness in recreational youth athletes during a season or competition has not been well studied. There is a need for increased knowledge in this population given 1) the scarcity of available data, 2) that most youth athletes participate in sport at recreational level, 3) results from adult and elite youth cohorts may not be extrapolated to this population owing to the difference between training load (intensity and volume), competition pressure, access to resources and travelling, and 4) to provide direction for illness prevention initiatives. In this context, this study aims to be the first to a) describe the epidemiological profile of weekly illness prevalence by sex, and b) describe the nature of the most common self-reported illnesses by sex in youth floorball players during a full 26-weeks season.

Methods

This prospective cohort study was part of a Sport Without Injury ProgrammE (SWIPE) cluster randomised controlled trial (RCT) in floorball evaluating the efficacy of the *Knee Control* injury prevention exercise programme (Clinical Trials registration NCT03309904). Representatives from the

For peer review only - http://bmjopen.bmj.com/site/about/guidelines.xhtml

Swedish Floorball Federation informed development of the *Knee Control* injury prevention exercise programme, and coaches (end-users) were involved in the RCT. The study was conducted in line with the Declaration of Helsinki, and approved by the Swedish Ethical Review Authority (Dnr 2017/294-31). Since the 15-minute warm-up training intervention was not believed to influence illness occurrences, we included all players in both the intervention and control group arms of the RCT for analysis in this sub-study. Youth male and female community level floorball players aged 12–17 years from two provinces of southern Sweden (Östergötland and Småland) registered to play floorball in a club during the 2017–2018 season (26 weeks) were included.[14 15]

To prospectively collect illness data, all players completed a weekly web-based survey during the season using the Oslo Sports Trauma Research Center (OSTRC) questionnaire on health problems.[16] If players answered anything else than the first option of the OSTRC questionnaire question 1, i.e. “full participation in floorball training and matches during the past week without health problems”, they were asked to indicate if their health problem was an injury/complaint or illness. Players who selected illness then answered a question about their main illness symptoms during the past week, and which day(s), if any, they had missed scheduled floorball training or matches due to illness (Table 1). Injury was the primary outcome of interest in the intervention study, and if a player reported an injury in the weekly survey they could not report a concurrent illness in the same week. Weekly surveys were administered each Sunday at 18:00 hours via a web-link through short messaging service (SMS) and email to the players, using the online survey software esMakerNX3 V3. If the players did not complete the survey on the same or the following day, two additional reminders were sent via SMS on the Tuesday and Thursday. All player reports were checked by a research coordinator (physiotherapist) for completeness and accuracy, and who contacted the player in case of any suspected errors in the registration. Player illness data were included for the full season or up until censoring, e.g. due to quitting floorball, or a season-ending injury.

For peer review only - http://bmjopen.bmj.com/site/about/guidelines.xhtml

Table 1: Weekly survey questions used to collect illness data during the 26-week season

Illness

Have you had any difficulties participating in normal floorball training during the past week due to health problems (such as pain, aches, tenderness, stiffness), injury or illness?[a]

- Full participation without health problems, injury or illness
- Full participation, but with health problems, injury or illness
- Reduced participation due to health problems, injury or illness
- Cannot participate due to health problems, injury or illness

Was your current health problem an injury/complaint or illness?

- Injury
- Illness

Select the option that best matches your major illness symptoms. (Enter using the drop-down list)

- Fever
- Feeling tired/feverish
- Swollen glands
- Sore throat
- Nasal congestion/runny nose/sneezing
- Coughing
- Difficulty breathing/tight airways
- Headache
- Nausea
- Vomiting
- Diarrhoea
- Constipation
- Fainting
- Skin rash/itchy skin lesions
- Irregular or abnormal heartbeats, chest pain/chest pain or discomfort with exercise
- Abdominal pain
- Other pain
- Numbness
- Nerve tingling/pain
- Anxiety
- Depression/feeling down
- Irritation
- Eye symptoms
- Ear symptoms
- Urogenital/gynaecological symptoms
- Other

[a] Question from the Oslo Sports Trauma Research Center questionnaire on health problems[16]

A total of 7503 weekly player reports were registered over the 26 weeks; 745 weekly reports were excluded because the player was absent from floorball the whole week due to reasons other than injury or illness (e.g. holiday). The final analysis thus comprised 6758 weekly player reports. The average weekly illness prevalence for the whole season was calculated by dividing the number of weeks a player had reported illness by the total number of weekly reports. Illness prevalence was

For peer review only

also calculated for each week of the season by dividing the number of players reporting an illness by the number of questionnaire responses for each week. If a player reported multiple illness episodes these were included as separate events. Illness present at the start of the season were included in prevalence calculations. According to the 2020 International Olympic Committee consensus recommendations, we grouped illness symptoms into clusters post study.[17] Sex differences in average weekly illness prevalence was compared with a prevalence rate ratio (PRR) and corresponding 95% CI, and proportion of reported illness symptoms with the chi-square test. Data were analysed using SPSS® 25.0 (IBM SPSS Statistics 2015).

Results

There were 471 players included in 47 teams. The mean age for 329 male and 142 female players was 13.3 (SD 1.0) and 13.7 (SD 1.5) years, respectively. The mean number of weekly reports per player was 14.4 (SD 7.8), with 13.5 (SD 8.1) for males and 16.3 (SD 6.8) for females. In total, 61% (n=288, 60% males vs 64% females) of players reported at least one illness during the season. Of the total, 6699 weekly reports with complete data for OSTRC question 1 (data were missing for 59 reports), 74% reports (76% male vs 71% female) indicated that players were able to fully participate in floorball without health problems, while for 12% (n=781) of all reports the player reported an illness. Among the 781 weeks with a reported illness, the player reported full floorball participation in 196 weeks (25%; 21% males vs 31% females), reduced participation in 202 weeks (26%; 26% in both males and females), and inability to participate in floorball due to illness in 383 weeks (49%; 53% males vs 43% females).

Illness prevalence during the season

The average weekly illness prevalence for the whole season was 12% (95% CI 10.8-12.3%) for all players, with slightly higher prevalence among females (13%, 95% CI 11.6-14.3%) than males (11%, 95% CI 9.9-11.7%), PRR 1.20 (95% CI 1.05-1.37, p=0.009).

For peer review only - http://bmjopen.bmj.com/site/about/guidelines.xhtml

The weekly illness prevalence ranged from 5% to 18% over the 26 weeks (4% to 23% female vs 5% - 17% male players). Illness prevalence was highest in calendar weeks 4 to 7 (peak winter weeks in January-February) and ranged from 15% - 18% in this period (20% - 23% females vs 12% - 17% males) (Figure 1).

<Insert "**Figure 1**: Weekly illness prevalence for all players and by sex during the 26-week floorball season" here>

Illness symptoms

The most common self-reported illness symptoms were fever (30%), sore throat (16%) and cough (14%), with no differences between sexes (Table 2). More female players (16% vs 3% males) reported difficulty of breathing/tight airways ($p<0.001$). Fainting was reported by 7% of the female players and no males (Table 2).

For peer review only - http://bmjopen.bmj.com/site/about/guidelines.xhtml

Table 2: Self-reported illness symptoms reported during the 26-week floorball season sorted by illness symptom cluster

**Illness symptom clusters**	**All**<br>**100% (n=773)**	**Female**<br>**100% (n=296)**	**Male**<br>**100% (n=477)**
Upper/lower respiratory (total)	*51.0 (394)*	*53.3 (158)*	*49.4 (236)*
- Sore throat	15.7 (121)	15.5 (46)	15.7 (75)
- Coughing	13.5 (104)	10.1 (30)	15.5 (74)
- Nasal congestion/runny nose/sneezing	12.8 (99)	10.8 (32)	14.0 (67)
- Difficulty breathing/tight airways	7.8 (60)	16.2 (48)	2.5 (12)
- Swollen glands	1.3 (10)	0.7 (2)	1.7 (8)
Non-specific illness (total)	*36.0 (27)*	*32.5 (96)*	*38.1 (182)*
- Fever	29.8 (230)	29.1 (86)	30.2 (144)
- Feeling tired/feverish	5.4 (42)	2.7 (8)	7.1 (34)
- Other pain	0.8 (6)	0.7 (2)	0.8 (4)
Neurological - Headache	*4.1 (32)*	*1.4 (4)*	*5.9 (28)*
Gastrointestinal (total)	*3.3 (25)*	*2.8 (8)*	*3.5 (17)*
- Nausea	1.8 (14)	1.4 (4)	2.1 (10)
- Vomiting	0.8 (6)	0.7 (2)	0.8 (4)
- Abdominal pain	0.4 (3)	0.7 (2)	0.2 (1)
- Diarrhoea	0.3 (2)	-	0.4 (2)
Cardiovascular - Fainting	*2.6 (20)*	*6.8 (20)*	-
Other - Unspecified	*2.2 (17)*	*2.0 (6)*	*2.3 (11)*
Otological - Ear symptoms	*0.4 (3)*	*0.7 (2)*	*0.2 (1)*
Psychological (total)	*0.4 (3)*	*0.6 (2)*	*0.2 (1)*
- Anxiety	0.3 (2)	0.3 (1)	0.2 (1)
- Irritation	0.1 (1)	0.3 (1)	-
Ophthalmological - Eye symptoms	*0.1 (1)*	-	*0.2 (1)*

Values are % of total weekly reports (number of weekly reports) with each reported symptom.

Weekly symptom prevalence for flu-like symptoms (fever, feeling tired/feverish) and upper/lower respiratory symptoms (swollen glands, sore throat, coughing, nasal congestion/runny nose/sneezing, difficulty breathing/tight airways) were high during January and February (weeks 4 – 7). Respiratory symptoms also peaked in mid-October (Figure 2).

For peer review only

For peer review only - http://bmjopen.bmj.com/site/about/guidelines.xhtml

<Insert "**Figure 2**: Weekly symptom prevalence for all players during the 26-week floorball season" here>

Discussion

The main findings of this study were that (1) 61% of youth floorball players reported at least one illness week during the season, with an average weekly illness prevalence of 12%, (2) 49% of all weekly reports with an illness indicated that the player was not able to participate in floorball (time loss), (3) illness prevalence was slightly higher among females, (4) upper and lower respiratory symptoms such as fever (30%), sore throat (16%) and cough (14%) were the most common, and (5) illness prevalence was highest in the peak winter months (late January/February) affecting 15% to 18% of the players in this period.

Illness prevalence

The average weekly illness prevalence was 12% and comparable with Norwegian youth elite athletes participating in technical sports (10%),[8] but higher than for Norwegian elite youth athletes participating in team sports (8-9%),[8 18] Dutch junior tennis players (6%),[10] and Irish elite youth track and field athletes (7%).[19] The Swedish floorball season is from September to March (Autumn and Winter months) when illness is more prevalent compared to summer months when athletics and tennis are played. Therefore, youth players can be exposed to different viruses in school or public places. Also, some established norms associated with team sport from sharing drink bottles, towels, equipment and locker rooms, and social behaviour such as hugging, shaking hands and celebrations can enhance transmission of air- and droplet-borne pathogens like influenza. Our data show that one in two illnesses reported led to complete time loss from the sport. Sports clubs can implement various behavioural, lifestyle, and medical intervention strategies to ensure that sporting

For peer review only - http://bmjopen.bmj.com/site/about/guidelines.xhtml

environments are safe spaces for sports participation, which become even more crucial during the current Novel Coronavirus pandemic.[9 20 21] Education of athletes, coaches, parent and support personnel about risk mitigation strategies for viral infections, including physical distancing to reduce the risk of transmission, cough and sneeze etiquette, wearing masks when symptomatic (coughing, sneezing) to reduce potentially infective respiratory droplets, regular hand hygiene, not sharing equipment such as drinking bottles, and strategies that facilitate good quality sleep and sleep hygiene practices.[9] Improved health literacy, including awareness of self-monitoring of respiratory symptoms (even if mild), could help to reduce the risk of spreading the infection to team members.[20] Medial strategies also include implementing illness prevention guidelines, illness surveillance and respiratory symptom screening, identifying at-risk and vulnerable individuals and implementing full preventative precautions during high-risk periods.[9]

Most common symptoms were fever (30%), and upper/lower respiratory symptoms such as sore throat (16%) and cough (14%). These symptoms peaked during the winter season in late January/February (week 4-7) indicating influenza could be one common cause for illness in our Swedish youth floorball players. The severity of an influenza season is affected by the circulating influenza strain, vaccine matching the circulating strains of the virus and vaccine uptake rates.[22] Two types of virus, namely Influenza A and B viruses, co-circulate and cause respiratory infections like the seasonal flu, with influenza B causing milder illness than influenza A.[23] Interestingly, the 2017-18 season in Sweden was dominated by the influenza B/Yamagata and the number of cases increased sharply in week 3 until the peak in week 7.[24] Influenza B/Yamagata affect children, youths and young adults,[25] and they typically recover at home without seeking medical care for influenza.[24] Consequently, the seasonal flu may have a severe impact on our cohort of athletes with half of all illness leading to total time loss from sport. From a public health point of view, vaccination is the primary measure to prevent influenza and reduce its impact on the population. In Sweden, influenza vaccination coverage for individuals aged under 65 years is only about 2%.[24] Countries like Australia have a higher influenza vaccine uptake with 20% of 6-13 years old and 21% of youths older than 13

For peer review only - http://bmjopen.bmj.com/site/about/guidelines.xhtml

For peer review only

years.[26] Although cases of influenza in our data were not confirmed by a laboratory test, our data call for implementation of general illness prevention measures e.g., regular handwashing, decrease contact with those with respiratory symptoms, and regular hydration.

Comparison between sexes

We observed a slightly higher weekly illness prevalence in female youth floorball players than males (13% vs 11%), and our findings concur with results from several different sports in Norwegian youth elite athletes.[8 18] Also in elite adult sports, female athletes appear to be more prone to illness,[9] yet the influence of sex on illness prevalence remains unclear in elite youth sports.[10-13] A noteworthy difference in illness symptom patterns was that more female players (16% vs 3% males) in our study reported difficulty of breathing/tight airways. One may speculate that sex differences in anatomy and physiology, and hormonal fluctuations, can partly explain this finding. Female airways are smaller in diameter, they have smaller lungs with fewer bronchioles (i.e. small diffusion surface), smaller maximum expiratory flow and weaker respiratory muscles[27] and may thus experience greater respiratory system limitations such as increased total work of breathing from high flow resistance.[28] Menstrual cycle-induced hormonal fluctuations can also influence the airway and symptoms (e.g. wheeze, shortness of breath).[29 30] Further, approximately one in 20 female players reported fainting yet no male players reported fainting. Women under the age of 35 years faint twice as often as their male counterparts. Although the exact cause was unclear, carrying the genetic variant and sex can increase the risk of fainting.[31]

Methodological considerations

Self-reported illness data rely on young players memory, and consequently, subject to recall bias. Additionally, self-reported illness reports are open to interpretation bias of the survey question. Use of prospective weekly reports limits the recall period to one week. Question responses were standardised with a list of symptoms to collect as accurate self-reported data as possible. Since the diagnosis was not available from self-report of illness, we followed the 2020 International Olympic

For peer review only - http://bmjopen.bmj.com/site/about/guidelines.xhtml

Committee methods for reporting of illness data;[17] by symptoms, and symptom clusters. Female players had a higher survey response rate than males (16 vs 14 weekly reports during the season). It is unknown whether there is a bias towards higher or lower response rates in weeks where the athlete had experienced a health problem, thus risking either over-or under-inflation of the illness prevalence rate. Owing to the skip logic application in our survey, response burden is low for those who report illness. The sample of female floorball players was smaller than that of male players and may be less robust. Because of the way the survey was structured, there is a possibility that illness data might be under-reported. Injury was the primary outcome of interest in the intervention study, and if a player reported an injury in the weekly survey they could not report a concurrent illness in the same week. Therefore, our illness prevalence numbers represent the minimum illness rates in the study cohort.

Conclusions

Our novel findings of the prevalence and pattern of illness in youth floorball may help direct prevention strategies. Flu-like symptoms dominated. Athletes, coaches, parents and support personnel need to be aware of risk of infections and educated about risk mitigation in viral infections, including risk mitigation strategies. Improved awareness of self-monitoring of respiratory symptoms could help reduce the risk of spreading the infection to team members.

Acknowledgements

The authors would like to thank Taru Tervo, PhD, School of Sport Sciences, Umeå University, and Emil Risberg, The Swedish Floorball Federation, for input on the study plan, surveys and research questions and administrative assistance. We also thank the participating clubs, coaches and players for their participation in the study. The Sport Without Injury ProgrammE (SWIPE) has been

For peer review only - http://bmjopen.bmj.com/site/about/guidelines.xhtml

established at Linköping University, Linköping, Sweden, through grants from the Swedish Research Council and the Swedish National Centre for Research in Sports.

Author contributions

NKPP analysed and interpreted the data and produced the first draft of the manuscript. MW, HL, IÅ and SS provided critical comments on the manuscript. MH conceptualised the study, interpreted the data and provided critical comments on the manuscript. All authors were involved in designing the study and approved the final draft for submission.

Data sharing statement

De-identified data may be shared upon reasonable request.

Patient and public involvement

The Swedish Floorball Federation and the Floorball Competence Center (Innebandyns Kompetenscentrum) at Umeå University were included in the planning and preparation of this study. Their involvement included input on the study plan, surveys and research questions, help with recruitment of participants, and plans for dissemination of the study results to participants and wider relevant communities. They had no influence on the analyses, interpretation of results or manuscript preparation.

Patient consent for publication

Not required.

Checklist and flow diagram for the appropriate reporting statement

STROBE SIIS checklist

For peer review only - http://bmjopen.bmj.com/site/about/guidelines.xhtml

Licence statement

I, the Submitting Author has the right to grant and does grant on behalf of all authors of the Work (as defined in the below author licence), an exclusive licence and/or a non-exclusive licence for contributions from authors who are: i) UK Crown employees; ii) where BMJ has agreed a CC-BY licence shall apply, and/or iii) in accordance with the terms applicable for US Federal Government officers or employees acting as part of their official duties; on a worldwide, perpetual, irrevocable, royalty-free basis to BMJ Publishing Group Ltd ("BMJ") its licensees and where the relevant Journal is co-owned by BMJ to the co-owners of the Journal, to publish the Work in BMJ Open and any other BMJ products and to exploit all rights, as set out in our licence.

The Submitting Author accepts and understands that any supply made under these terms is made by BMJ to the Submitting Author unless you are acting as an employee on behalf of your employer or a postgraduate student of an affiliated institution which is paying any applicable article publishing charge ("APC") for Open Access articles. Where the Submitting Author wishes to make the Work available on an Open Access basis (and intends to pay the relevant APC), the terms of reuse of such Open Access shall be governed by a Creative Commons licence – details of these licences and which Creative Commons licence will apply to this Work are set out in our licence referred to above.

Figure legends

Figure 1: Weekly illness prevalence for all players and by sex during the 26-week floorball season

Figure 2: Weekly symptom prevalence for all players during the 26-week floorball season

For peer review only - http://bmjopen.bmj.com/site/about/guidelines.xhtml

References

1. Raysmith B, Drew M. Performance success or failure is influenced by weeks lost to injury and illness in elite Australian track and field athletes: a 5-year prospective study. *J Sci Med Sport* 2016;19(10):778-83.

2. Schwellnus M. The Olympic textbook of medicine in sport: John Wiley & Sons 2009:344-65.

3. Van Tonder A, Schwellnus M, Swanevelder S, et al. A prospective cohort study of 7031 distance runners shows that 1 in 13 report systemic symptoms of an acute illness in the 8-12 day period before a race, increasing their risk of not finishing the race 1.9 times for those runners who started the race: SAFER study IV. *Br J Sports Med* 2016;50(15):939-45.

4. Timpka T, Jacobsson J, Bargoria V, et al. Preparticipation predictors for championship injury and illness: cohort study at the Beijing 2015 International Association of Athletics Federations World Championships. *Br J Sports Med* 2017;51(4):271-76.

5. Friman G, Wesslen L. nfections and exercise in high-performance athletes. *Immunol Cell Biol* 2000;78(5):510-22.

6. Tseng GS, Hsieh CY, Hsu CT, et al. Myopericarditis and exertional rhabdomyolysis following an influenza A (H3N2) infection. *BMC Infect Dis* 2013;13:283.

7. Weidner T, Sevier T. Sport, exercise, and the common cold. *J Athl Train* 1996;31(2):154-59.

8. Moseid C, Myklebust G, Fagerland M, et al. The prevalence and severity of health problems in youth elite sports: A 6-month prospective cohort study of 320 athletes. *Scand J Med Sci Sports* 2018;28(4):1412-23.

9. Schwellnus M, Soligard T, Alonso J-M, et al. How much is too much? (Part 2) International Olympic Committee consensus statement on load in sport and risk of illness. *Br J Sports Med* 2016;50(17):1043-52.

10. Pluim B, Loeffen F, Clarsen B, et al. A one-season prospective study of injuries and illness in elite junior tennis. *Scand J Med Sci Sports* 2016;26(5):564-71.

For peer review only - http://bmjopen.bmj.com/site/about/guidelines.xhtml

11. Ruedl G, Schobersberger W, Pococco E, et al. Sport injuries and illnesses during the first Winter Youth Olympic Games 2012 in Innsbruck, Austria. *Br J Sports Med* 2012;46(15):1030-7.

12. Steffen K, Moseid C, Engebretsen L, et al. Sports injuries and illnesses in the Lillehammer 2016 Youth Olympic Winter Games. *Br J Sports Med* 2017;51(1):29-35.

13. Steffen K, Soligard T, Mountjoy M, et al. How do the new Olympic sports compare with the traditional Olympic sports? Injury and illness at the 2018 Youth Olympic Summer Games in Buenos Aires, Argentina. *Br J Sports Med* 2020;54(3):168-75.

14. Åkerlund I, Waldén M, Sonesson S, et al. Forty-five per cent lower acute injury incidence but no effect on overuse injury prevalence in youth floorball players (aged 12–17 years) who used an injury prevention exercise programme: two-armed parallel-group cluster randomised controlled trial. *Br J Sports Med* 2020;54(17):1028-35.

15. Perera N, Åkerlund I, Hägglund M. Motivation for sports participation, injury prevention expectations, injury risk perceptions and health problems in youth floorball players. *Knee Surg Sports Traumatol Arthrosc* 2019;27(11):3722-32.

16. Clarsen B, Rønsen O, Myklebust G, et al. The Oslo Sports Trauma Research Center questionnaire on health problems: a new approach to prospective monitoring of illness and injury in elite athletes. *Br J Sports Med* 2014;48(9):754-60.

17. Bahr R, Clarsen B, Derman W, et al. International Olympic Committee consensus statement: methods for recording and reporting of epidemiological data on injury and illness in sport 2020 (including STROBE Extension for Sport Injury and Illness Surveillance (STROBE-SIIS)). *Br J Sports Med* 2020;54(7):372-89.

18. Bjørndal C, Bache-Mathiesen L, Gjesdal S, et al. An examination of training load, match activities, and health problems in Norwegian youth elite handball players over one competitive season. *Front Sports Act Living* 2021;3(36)

For peer review only

For peer review only - http://bmjopen.bmj.com/site/about/guidelines.xhtml

19. Carragher P, Rankin A, Edouard P. A one-season prospective study of illnesses, acute, and overuse injuries in elite youth and junior track and field athletes. *Front Sports Act Living* 2019;1(13)

20. Hughes D, Saw R, Perera N, et al. The Australian Institute of Sport framework for rebooting sport in a COVID-19 environment. *J Sci Med Sport* 2020;23(7):639-63.

21. Schwellnus M, Janse van Rensburg C, Bayne H, et al. Team illness prevention strategy (TIPS) is associated with a 59% reduction in acute illness during the Super Rugby tournament: a control–intervention study over 7 seasons involving 126 850 player days. *Br J Sports Med* 2020;54(4):245-49.

22. Moa A, Trent M, Menzies R. Severity of the 2019 influenza season in Australia-a comparison between 2017 and 2019 H3N2 influenza seasons. *Global Biosecurity* 2019;1(3)

23. Centers for Disease Control and Prevention. Types of influenza viruses. Centers for Disease Control and Prevention, National Center for Immunization and Respiratory Diseases (NCIRD), 19 November 2019. Availabe: https://www.cdc.gov/flu/about/viruses/types.htm [Accessed October 19, 2020]

24. Brytting M, Dahl H, Wiman Å, et al Influenza in Sweden 2017–2018. Public Health Agency of Sweden, Stockholm, Sweden 2018.

25. Moa A, Muscatello D, Turner RM et al. Epidemiology of influenza B in Australia: 2001-2014 influenza seasons. *Influenza Other Respir Viruses* 2017;11(2):102-09.

26. Rhodes A. Flu vaccination: Perspectives of Australian parents: Australian Child Health Poll, The Royal Childrens Hospital Melbourne, May 2017 (Poll 6A).

27. LoMauro A, Aliverti A. Sex differences in respiratory function. *Breathe (Sheff)* 2018;14(2):131-40.

28. Guenette J, Querido J, Eves D, et al. Sex differences in the resistive and elastic work of breathing during exercise in endurance-trained athletes. *Am J Physiol Regul Integr Comp Physiol* 2009;297(1):R166-75.

For peer review only - http://bmjopen.bmj.com/site/about/guidelines.xhtml

29. Graziottin A, Serafini A. Perimenstrual asthma: from pathophysiology to treatment strategies. *Multidiscip Respir Med* 2016;11(1):30.

30. Macsali F, Svanes C, Sothern R, et al. Menstrual cycle and respiratory symptoms in a general Nordic-Baltic population. *Am J Respir Crit Care Med* 2013;187(4):366-73.

31. Hadji-Turdeghal K, Andreasen L, Hagen C, et al. Genome-wide association study identifies locus at chromosome 2q32.1 associated with syncope and collapse. *Cardiovasc Res* 2019;116(1):138-48.

For peer review only

For peer review only - http://bmjopen.bmj.com/site/about/guidelines.xhtml

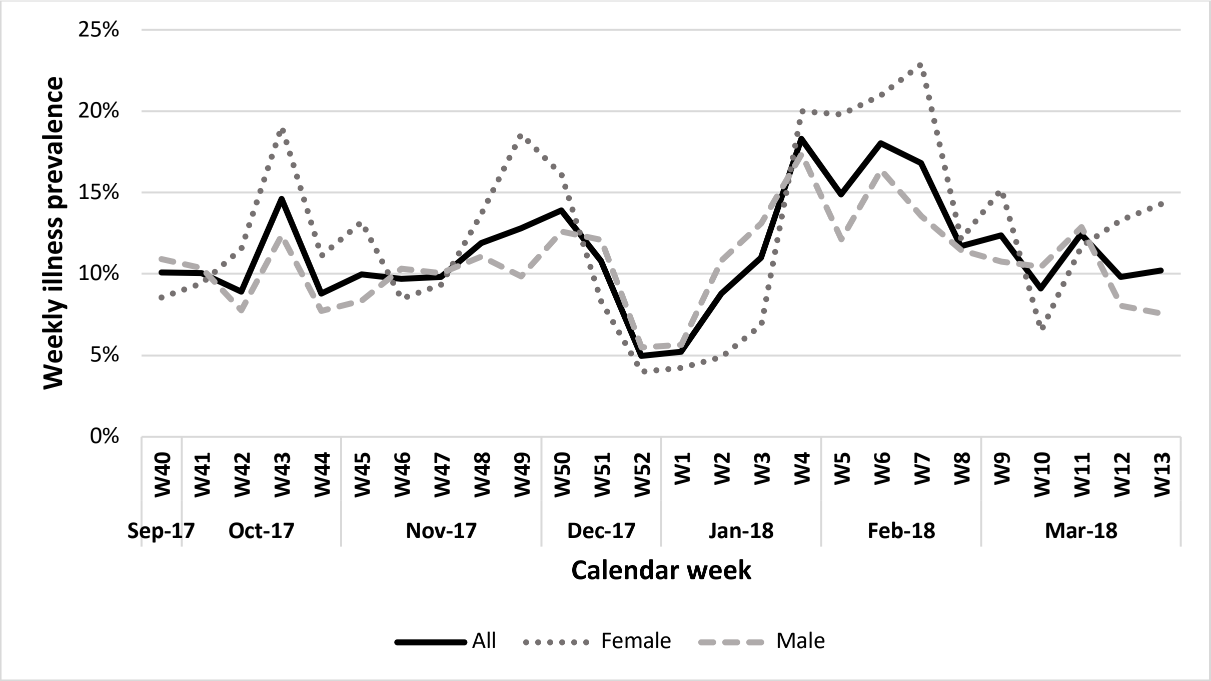

Figure 1: Weekly illness prevalence for all players and by sex during the 26-week floorball season Many teams had reduced training during weeks 52 and 1 due to Christmas and New year holidays.

For peer review only

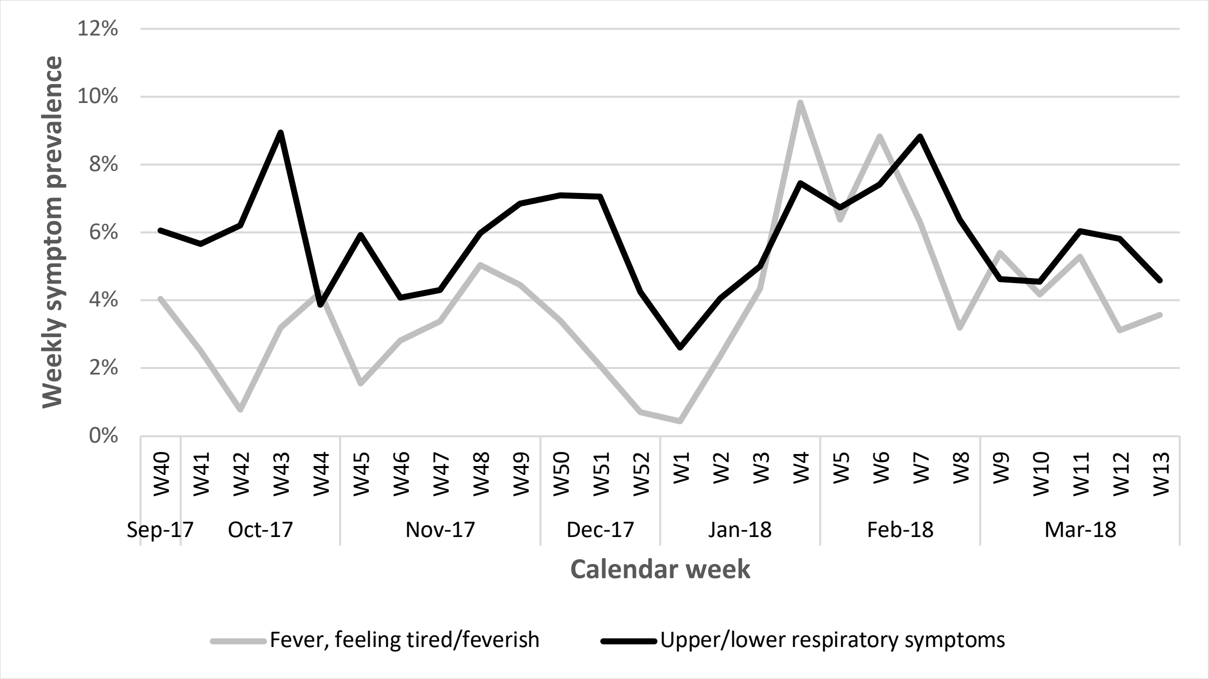

Key: Upper/lower respiratory symptoms include swollen glands, sore throat, coughing, nasal congestion/runny nose/sneezing, difficulty breathing/tight airways

Figure 2: Weekly symptom prevalence for all players during the 26-week floorball season

For peer review only

Appendix 3: STROBE-SIIS (Sports Injury and Illness Surveillance) Statement 1.0—Checklist of items for the reporting of observational studies on injury and illness in sports

Item	Item No	Recommendation from the STROBE Statement	STROBE-SIIS Extension +	Reported on Page #
**Title and abstract**	1	(*a*) Indicate the study's design with a commonly used term in the title or the abstract		1-2
		(*b*) Provide in the abstract an informative and balanced summary of what was done and what was found	SIIS-1.1. Include information on the sport, athlete population (sex, age, geographic region) and level of competition.<br>SIIS-1.2. Include the duration of observation (e.g. one season, one year, multiple years).	2
**Introduction**				
Background/rationale	2	Explain the scientific background and rationale for the investigation being reported		4
Objectives	3	State specific objectives, including any pre-specified hypotheses	SIIS-3.1. State whether study was registered. Identify the registration number and database used.<br>SIIS-3.2. State the specific purpose of your study (e.g. to describe the injury burden associated with Olympic level rowing)	4
**Methods**				
Study design	4	Present key elements of study design early in the paper	SIIS-4.1. Clearly specify which health problems are being observed.	5-6

For peer review only

Bahr R, *et al. Br J Sports Med* 2020; 54:372–389. doi: 10.1136/bjsports-2019-101969

Item	Item No	Recommendation from the STROBE Statement	STROBE-SIIS Extension +	
			SIIS-4.2. State explicitly which approach was used to record the health problem data, including all outcome measures or tools	5-6
			SIIS-4.3. State explicitly which coding system was used to classify the health problems (e.g. OSIICS, SMDCS, ICD, etc.)	5-7
			SIIS-4.4. Where relevant, clearly describe how athletes were categorised. Variables to consider could include the type of athlete and/or sport, the environment in which the sport occurs (e.g. type of course or playing area), the typical duration of the sport, the degree of physical contact permitted in the sport, and the equipment permitted.	5
Setting	5	Describe the setting, locations, and relevant dates, including periods of recruitment, exposure, follow-up, and data collection	SIIS-5.1. Describe the location, level of play, dates of observation and data collection methods (i.e. who, what, where).	5
			SIIS-5.2. Specify the dates of the surveillance period and how the data were handled when the study covered more than one season/calendar year.	5
			SIIS-5.3. Define whether the health problem data were collected prospectively or retrospectively.	4-5

For peer review only

For peer review only - http://bmjopen.bmj.com/site/about/guidelines.xhtml

Item	Item No	Recommendation from the STROBE Statement	STROBE-SIIS Extension +	Reported on Page #
Participants	6	(*a*) *Cohort study*—Give the eligibility criteria, and the sources and methods of selection of participants. Describe methods of follow-up<br>*Case-control study*—Give the eligibility criteria, and the sources and methods of case ascertainment and control selection. Give the rationale for the choice of cases and controls<br>*Cross-sectional study*—Give the eligibility criteria, and the sources and methods of selection of participants	SIIS-6.1. Define the population of athletes and how they were selected and recruited.	5
		(*b*) *Cohort study*—For matched studies, give matching criteria and number of exposed and unexposed<br>*Case-control study*—For matched studies, give matching criteria and the number of controls per case		
Variables	7	Clearly define all outcomes, exposures, predictors, potential confounders, and effect modifiers. Give diagnostic criteria, if applicable	SIIS-7.1. Justify why you measured your primary and secondary outcomes of interest in the specific way chosen.<br>SIIS-7.2. Describe the method for identifying your health problem outcome of interest.	6-7
Data sources/ measurement	8*	For each variable of interest, give sources of data and details of methods of assessment (measurement). Describe	SIIS-8.1. Specify who collected/reported the data for the	5

For peer review only

Bahr R, *et al. Br J Sports Med* 2020; 54:372–389. doi: 10.1136/bjsports-2019-101969

Item	Item No	Recommendation from the STROBE Statement	STROBE-SIIS Extension +	Reported on Page #
		comparability of assessment methods if there is more than one group	study and their qualifications (e.g. qualified doctor, data analyst, etc.).	
			SIIS-8.2. Specify who coded the data for the study and their qualifications (e.g. qualified doctor, data analyst, etc.). In many instances, this will not be the same as SIIS-8.1.	5
			SIIS-8.3. Specify the direct methods used to collect the data, and the use of physical documents or an electronic tools. If extracting information from existing sources, specify the data source.	5
			SIIS-8.4. Specify the timing of and window for data collection (e.g. day health problem occurred or following day). Specify the frequency of data collection (e.g. daily, weekly, monthly).	5
			SIIS-8.5. Report the duration of surveillance (e.g. tournament, season, whole year, playing career).	5
Bias	9	Describe any efforts to address potential sources of bias	SIIS-9.1. Clearly report any validation or reliability assessment of the data collection of tools.	
			SIIS-9.2. Formally acknowledge any potential biases in associated with the data collection method (e.g. self-	13

For peer review only

Bahr R, *et al. Br J Sports Med* 2020; 54:372–389. doi: 10.1136/bjsports-2019-101969

Item	Item No	Recommendation from the STROBE Statement	STROBE-SIIS Extension +	Reported on Page #
			report, recall bias, reporting by non-medically trained staff, etc.)	
Study size	10	Explain how the study size was arrived at		5, 10
Quantitative variables	11	Explain how quantitative variables were handled in the analyses. If applicable, describe which groupings were chosen and why	SIIS-11.1 Explain in detail how multiple injuries/illness episodes are handled both in individual athletes and across athletes/surveillance periods.	7
			SIIS-11.2. Specify how injury severity was calculated.	N/A
Statistical methods	12	(*a*) Describe all statistical methods, including those used to control for confounding	SIIS-12.1. Specify how exposure to risk has been adjusted for and specify the units (e.g. per participant, per athlete exposure, etc.).	6-7
			SIIS-12.2 Specify how relevant risk measures (incidence, prevalence, etc.) were calculated.	6-7
			SIIS-12.3. When relevant to the study aim, specify how injury burden was calculated and analysed.	N/A
		(*b*) Describe any methods used to examine subgroups and interactions		6-7
		(*c*) Explain how missing data were addressed	SIIS-12.4. For studies reporting multiple health problems, state clearly how these were handled (e.g. time to the first injury only, ignoring	7

For peer review only

Bahr R, *et al. Br J Sports Med* 2020; 54:372–389. doi: 10.1136/bjsports-2019-101969

Item	Item No	Recommendation from the STROBE Statement	STROBE-SIIS Extension +	Reported on Page #
			subsequent return to play and re-injuries, or modelling of all injuries).	
			SIIS-12.5. Explain how/if athletes not included at outset (e.g. those already injured) were handled in the analyses.	7
		(*d*) *Cohort study*—If applicable, explain how loss to follow-up was addressed<br>*Case-control study*—If applicable, explain how matching of cases and controls was addressed<br>*Cross-sectional study*—If applicable, describe analytical methods taking account of sampling strategy	SIIS-12.6. In longitudinal studies, it is particularly important to explain how athlete follow-up has been managed. For example, what happened if a player was transferred to another team or has been censored (for those no longer part of the study due to removal during the observation period). Censoring can occur when athletes are removed due to transfer out of the team/study, injury/illness or due to study design.	6
		(*e*) Describe any sensitivity analyses		N/A
**Results**				
Participants	13*	(a) Report numbers of individuals at each stage of study—e.g. numbers potentially eligible, examined for eligibility, confirmed eligible, included in the study, completing follow-up, and analysed	SIIS-13.1. Clearly state the number of athletes followed-up, the number (and %) of those with the health problem and the number of problems reported among them (a median number of problems per affected athlete could be useful).	7
			SIIS-13.2. For studies over multiple seasons/years, report the total	N/A

For peer review only

Bahr R, *et al. Br J Sports Med* 2020; 54:372–389. doi: 10.1136/bjsports-2019-101969

Item	Item No	Recommendation from the STROBE Statement	STROBE-SIIS Extension +	Reported on Page #
			numbers of health problems for each year and numbers common to each period.	
		(b) Give reasons for non-participation at each stage	SIIS-13.3. Report how athletes removed (e.g. due to transfer of teams or time-out due to injury or illness) impact upon data at key data collection/reporting points, ideally with a flow diagram	
Descriptive data	14*	(a) Give characteristics of study participants (e.g. demographic, clinical, social) and information on exposures and potential confounders	SIIS-14.1. Include detail on the level of competition being observed (e.g. by age levels, skill level, sex, etc.).	5,7
		(b) Indicate number of participants with missing data for each variable of interest		
		(c) *Cohort study*—Summarise follow-up time (e.g. average and total amount)		
Outcome data	15*	*Cohort study*—Report numbers of outcome events or summary measures over time	SIIS-15.1. In many observational studies, individuals will sustain more than one health problem over the surveillance period. Take care to ensure descriptive data representing both the number of health problems and the number of athletes affected. It is important to represent effectively both the analysis and reporting of correct units for frequency data, i.e. the % of affected	7-9

For peer review only

Bahr R, *et al. Br J Sports Med* 2020; 54:372–389. doi: 10.1136/bjsports-2019-101969

Item	Item No	Recommendation from the STROBE Statement	STROBE-SIIS Extension +	
			athletes or the % of injuries, body regions, etc.	
		Case-control study—Report numbers in each exposure category, or summary measures of exposure		
		Cross-sectional study—Report numbers of outcome events or summary measures		
Main results	16	(*a*) Give unadjusted estimates and, if applicable, confounder-adjusted estimates and their precision (e.g. 95% confidence interval). Make clear which confounders were adjusted for and why they were included	SIIS-16.1. Report exposure-adjusted incidence or prevalence measures with appropriate confidence intervals when presenting risk measures.<br>SIIS-16.2. Report details of interest, such as mode of onset	7-10
		(*b*) Report category boundaries when continuous variables were categorized		
		(*c*) If relevant, consider translating estimates of relative risk into absolute risk for a meaningful time period		
Other analyses	17	Report other analyses done—e.g. analyses of subgroups and interactions, and sensitivity analyses	SIIS-17.1 Report injury diagnosis information, including region and tissue type in tabular form.	9
**Discussion**				
Key results	18	Summarise key results with reference to study objectives		10,14
Limitations	19	Discuss limitations of the study, taking into account sources of potential bias or	SIIS-19.1. Discuss limitations in the data collection and coding procedures adopted, including in	13

For peer review only - http://bmjopen.bmj.com/site/about/guidelines.xhtml

Item	Item No	Recommendation from the STROBE Statement	STROBE-SIIS Extension +	Reported on Page #
		imprecision. Discuss both direction and magnitude of any potential bias	relation to any risk measures calculated.	
Interpretation	20	Give a cautious overall interpretation of results considering objectives, limitations, multiplicity of analyses, results from similar studies, and other relevant evidence		10-14
Generalisability	21	Discuss the generalisability (external validity) of the study results	SIIS-21.1. Discuss the generalizability of the athlete study population, and health problem sub-groups of interest, to broader athlete groups.	10-14
**Other information**				
Funding	22	Give the source of funding and the role of the funders for the present study and, if applicable, for the original study on which the present article is based		3
Ethics	23		SIIS-23.1. Outline how individual athlete data privacy and confidentiality considerations were addressed, in line with the Declaration of Helsinki.	5

Note: The STROBE-SIIS checklist with additional sports epidemiology annotations should be used in conjunction with the original STROBE guideline (freely available on the Web sites of PLoS Medicine at http://www.plosmedicine.org/, Annals of Internal Medicine at http://www.annals.org/, and Epidemiology at http://www.epidem.com/). Information on the STROBE Initiative is available at www.strobe-statement.org.

+For brevity, the phrase health problem is used here to encompass both injury and illness.

For peer review only - http://bmjopen.bmj.com/site/about/guidelines.xhtml

*Give information separately for cases and controls in case-control studies and, if applicable, for exposed and unexposed groups in cohort and cross-sectional studies.

^^ Where there is a blank cell in this column, there are no specific additional reporting requirements for sports injury and illness surveillance over what is already covered in the original STROBE checklist.

Reference

1. Orchard O, Meeuwisse W, Derman W, et al. Refinement and presentation of the Calgary Sport Medicine Diagnostic Coding System (SMDSC) and the Orchard Sport Injury & Illness Classification System (OSIICS). *Br J Sports Med* In preparation

For peer review only - http://bmjopen.bmj.com/site/about/guidelines.xhtml

BMJ Open

Illness prevalence and symptoms in youth floorball players – a one-season prospective cohort study involving 471 players.

Journal:	*BMJ Open*
Manuscript ID	bmjopen-2021-051902.R1
Article Type:	Original research
Date Submitted by the Author:	19-Oct-2021
Complete List of Authors:	Panagodage Perera, Nirmala; Linköping University, Department of Health, Medicine and Caring Sciences, Unit of Physiotherapy, Sport Without Injury ProgrammE (SWIPE); Australian Institute of Sport<br>Waldén, Markus; Linköping University, Department of Health, Medicine and Caring Sciences, Unit of Physiotherapy, Sport Without Injury ProgrammE (SWIPE); Hässleholm-Kristianstad Hospitals, Department of Orthopaedics<br>Lindblom, Hanna ; Linköping University, Department of Health, Medicine and Caring Sciences, Unit of Physiotherapy, Sport Without Injury ProgrammE (SWIPE)<br>Åkerlund, Ida; Linköping University, Department of Health, Medicine and Caring Sciences, Unit of Physiotherapy, Sport Without Injury ProgrammE (SWIPE)<br>Sonesson, Sofi; Linköping University, Department of Health, Medicine and Caring Sciences, Unit of Physiotherapy, Sport Without Injury ProgrammE (SWIPE)<br>Hagglund, Martin; Linköping University, Department of Health, Medicine and Caring Sciences, Unit of Physiotherapy, Sport Without Injury ProgrammE (SWIPE)
**Primary Subject Heading**:	Sports and exercise medicine
Secondary Subject Heading:	Epidemiology
Keywords:	EPIDEMIOLOGY, SPORTS MEDICINE, PUBLIC HEALTH

SCHOLARONE™
Manuscripts

I, the Submitting Author has the right to grant and does grant on behalf of all authors of the Work (as defined in the below author licence), an exclusive licence and/or a non-exclusive licence for contributions from authors who are: i) UK Crown employees; ii) where BMJ has agreed a CC-BY licence shall apply, and/or iii) in accordance with the terms applicable for US Federal Government officers or employees acting as part of their official duties; on a worldwide, perpetual, irrevocable, royalty-free basis to BMJ Publishing Group Ltd ("BMJ") its licensees and where the relevant Journal is co-owned by BMJ to the co-owners of the Journal, to publish the Work in this journal and any other BMJ products and to exploit all rights, as set out in our licence.

The Submitting Author accepts and understands that any supply made under these terms is made by BMJ to the Submitting Author unless you are acting as an employee on behalf of your employer or a postgraduate student of an affiliated institution which is paying any applicable article publishing charge ("APC") for Open Access articles. Where the Submitting Author wishes to make the Work available on an Open Access basis (and intends to pay the relevant APC), the terms of reuse of such Open Access shall be governed by a Creative Commons licence – details of these licences and which Creative Commons licence will apply to this Work are set out in our licence referred to above.

Other than as permitted in any relevant BMJ Author's Self Archiving Policies, I confirm this Work has not been accepted for publication elsewhere, is not being considered for publication elsewhere and does not duplicate material already published. I confirm all authors consent to publication of this Work and authorise the granting of this licence.

For peer review only

Illness prevalence and symptoms in youth floorball players – a one-season prospective cohort study involving 471 players.

Perera NKP, [1,2,3] Waldén M,[2,4,5] Lindblom H, [1,2] Åkerlund I, [1,2] Sonesson S, [1,2] Hägglund M[1,2]

[1] Unit of Physiotherapy, Department of Health, Medicine and Caring Sciences (HMV), Linköping University, Linköping, Sweden

[2] Sport Without Injury ProgrammE (SWIPE), Department of Health, Medicine and Caring Sciences (HMV), Linköping University, Linköping, Sweden

[3] Sport Medicine, The Australian Institute of Sport, Burce, ACT, Australia

[4] Unit of Community Medicine, Department of Health, Medicine and Caring Sciences (HMV), Linköping University, Linköping, Sweden

[5] Department of Orthopaedics, Hässleholm-Kristianstad Hospitals, Hässleholm, Sweden

Nirmala Perera perera.nk@outlook.com

Markus Waldén markus.walden@telia.com

Hanna Lindblom hanna.lindblom@liu.se

Ida Åkerlund ida.akerlund@liu.se

Sofi Sonesson sofi.sonesson@liu.se

Martin Hägglund (corresponding author) martin.hagglund@liu.se Unit of Physiotherapy, Department of Health, Medicine and Caring Sciences (HMV), Linköping University, 581 83 Linköping, Sweden. Tel +46 733347704

Keywords: Sport, Illness, Youth, Epidemiology

Word count: 2523

For peer review only

Abstract

Objective: To describe weekly illness prevalence and illness symptoms by sex in youth floorball players during one season.

Design: Prospective cohort study

Setting: Players who were registered to play community level floorball during the 2017–2018 season (26 weeks) in two provinces in southern Sweden.

Participants: 471 youth players aged 12–17 years. Mean (SD) age for 329 male players 13.3 (1.0) years and 142 female players 13.7 (1.5) years.

Primary and secondary outcome measures: Weekly self-reported illness prevalence and illness symptoms, arranged according to the 2020 International Olympic Committee's consensus recommendations.

Results: 61% of youth floorball players reported at least one illness week during the season, with an average weekly illness prevalence of 12% (95% CI 10.8–12.3%). The prevalence was slightly higher among females (13%, 95% CI 11.6–14.3%) than males (11%, 95% CI 9.9–11.7%), prevalence rate ratio 1.20 (95% CI 1.05–1.37, p=0.009). In total, 49% (53% male, 43% female) of illness reports indicated that the player could not participate in floorball (time loss), with a mean (SD) absence of 2.0 (1.7) days per illness week. Fever (30%), sore throat (16%) and cough (14%) were the most common symptoms. Female players more often reported difficulty in breathing/tight airways and fainting, and male players more often reported coughing, feeling tired/feverish and headache. Illness prevalence was highest in the peak winter months (late January/February) reaching 15–18% during this period.

Conclusions: Our novel findings of the illness prevalence and symptoms in youth floorball may help direct prevention strategies. Athletes, coaches, parents and support personnel need to be educated about risk mitigation strategies.

For peer review only

For peer review only - http://bmjopen.bmj.com/site/about/guidelines.xhtml

Trial registration: Clinical Trials Registration NCT03309904

Article Summary – Strengths and limitations of this study

- The first study to evaluate self-reported prevalence, seasonal variations and illness symptom clusters by sex according to the 2020 International Olympic Committee's methods for recording and reporting epidemiological data consensus recommendations.
- Illness data from a large sample of male and female recreational youth floorball players over a 26-week period.
- Self-reported illness data may be subject to recall and interpretation bias, and the actual diagnosis was not available.
- Injury was the primary outcome of interest in the intervention study; players who reported an injury in the weekly survey could not report a concurrent illness during the same week, and illness data thus represents minimum illness prevalence rates in the study cohort.

Funding statement

The Sport Without Injury ProgrammE is funded by the Swedish Research Council (2015-02414 and 2018-03135) and the Swedish Research Council for Sport Science (P2018-0167).

Competing interests statement

None declared.

For peer review only - http://bmjopen.bmj.com/site/about/guidelines.xhtml

Introduction

Acute illness presents a significant health burden to athletes, interfering with their training, reducing sport performance and causing time loss from competitions,[1-3] and may increase the risk of subsequent injuries.[4] Strenuous exercise during acute infection can increase the risk of serious medical complications, including sudden death.[2 5-7] Performance impairments from upper respiratory tract illness may also last for 2–4 days after complete recovery.[2] Infectious illness can reduce muscle strength and impair motor coordination,[7] decrease aerobic and endurance capacity[5] and alter metabolic function[7] during and/or after the infection.

In elite youth sports, illness prevalence is higher among athletes who participate in endurance sports (23%) than those who participate in technical (10%) or team (8%) sports.[8] In elite adult sports, illness is more common among female athletes and those who participate in outdoor winter sports;[9] however, this relationship is unclear in elite youth sports.[10-13] The incidence of illness among recreational youth athletes during a season or competition has not been well studied. There is a need for increased knowledge about this population, given: 1) the scarcity of available data, 2) that most youth athletes participate in sport at recreational level, 3) it may not be appropriate to extrapolate the results from adult and elite youth cohorts to this population owing to the differences in training load (intensity and volume), competition pressure, access to resources and travelling, and 4) to provide direction for illness prevention initiatives. In this context, this study aims to be the first to: a) describe the epidemiological profile of weekly illness prevalence by sex, and b) describe self-reported illnesses symptoms by sex among youth floorball players during a full 26-week season.

Methods

This prospective cohort study was part of a Sport Without Injury ProgrammE (SWIPE) cluster randomised controlled trial (RCT) in floorball evaluating the efficacy of the *Knee Control* injury prevention exercise programme (Clinical Trials registration NCT03309904). Representatives from the

For peer review only

For peer review only - http://bmjopen.bmj.com/site/about/guidelines.xhtml

Swedish Floorball Federation informed the development of the *Knee Control* injury prevention exercise programme, and coaches (end-users) were involved in the RCT. The study was conducted in line with the Declaration of Helsinki, and approved by the Swedish Ethical Review Authority (Dnr 2017/294-31). Written informed consent was collected from all participating players, and from legal guardians for players below 15 years of age. Since the 15-minute warm-up training intervention was not believed to influence illness occurrences, we included all players in both the intervention and control group arms of the RCT for analysis in this sub-study. Youth male and female community-level floorball players aged 12–17 years from two provinces in southern Sweden (Östergötland and Småland) registered to play floorball in a club during the 2017–2018 season (26 weeks) were included.[14 15] Floorball is a winter sport played indoors on a playing surface made of rubber or parquet, using a plastic stick and light plastic ball on a 40 x 20 m pitch. There are five field players and one goalkeeper on the court in each team with free substitutes.

To prospectively collect illness data, all players completed a weekly web-based survey during the season using the Oslo Sports Trauma Research Center (OSTRC) questionnaire on health problems.[16] If players answered anything other than the first option on the OSTRC questionnaire question 1, i.e. “full participation in floorball training and matches during the past week without health problems”, they were asked to indicate whether their health problem was an injury/complaint or illness. Players who selected illness then answered a question about their main illness symptoms during the past week, and which day(s), if any, they had missed scheduled floorball training or matches due to illness (Table 1). Injury was the primary outcome of interest in the intervention study, and if a player reported an injury in the weekly survey they could not report a concurrent illness during the same week. Weekly surveys were administered each Sunday at 18:00 hours via a web-link sent via short messaging service (SMS) and email to the players, using the online survey software esMakerNX3 V3. If the players did not complete the survey on the same or the following day, two additional reminders were sent via SMS on the Tuesday and Thursday. All player reports were checked by a research coordinator (physiotherapist) for completeness and accuracy, and this coordinator

For peer review only - http://bmjopen.bmj.com/site/about/guidelines.xhtml

contacted the player in case of any suspected errors in the registration. Player illness data were included for the full season or up until censoring, e.g. due to quitting floorball, or a season-ending injury.

Table 1: Weekly survey questions used to collect illness data during the 26-week season

**Illness**
Have you had any difficulties participating in normal floorball training during the past week due to health problems (such as pain, aches, tenderness, stiffness), injury or illness?[a]<br>– Full participation without health problems, injury or illness<br>– Full participation, but with health problems, injury or illness<br>– Reduced participation due to health problems, injury or illness<br>– Could not participate due to health problems, injury or illness
Was your current health problem an injury/complaint or illness?<br>– Injury<br>– Illness
Select the option that best matches your major illness symptoms. (Enter using the drop-down list)<br>– Fever<br>– Feeling tired/feverish<br>– Swollen glands<br>– Sore throat<br>– Nasal congestion/runny nose/sneezing<br>– Coughing<br>– Difficulty breathing/tight airways<br>– Headache<br>– Nausea<br>– Vomiting<br>– Diarrhoea<br>– Constipation<br>– Fainting<br>– Skin rash/itchy skin lesions<br>– Irregular or abnormal heartbeats, chest pain or discomfort with exercise<br>– Abdominal pain<br>– Other pain<br>– Numbness<br>– Nerve tingling/pain<br>– Anxiety<br>– Depression/feeling down<br>– Irritation<br>– Eye symptoms<br>– Ear symptoms<br>– Urogenital/gynaecological symptoms<br>– Other
Which day(s) during the past week did you miss scheduled floorball training or matches due to illness?

[a] Question from the Oslo Sports Trauma Research Center questionnaire on health problems[16]

For peer review only

For peer review only - http://bmjopen.bmj.com/site/about/guidelines.xhtml

123 A total of 7503 weekly player reports were registered over the 26 weeks; 745 weekly reports were excluded because the player was absent from floorball for the whole week due to reasons other than injury or illness (e.g. holiday). The final analysis thus comprised 6758 weekly player reports. The average weekly illness prevalence for the whole season was calculated by dividing the number of weeks a player had reported illness by the total number of weekly reports. Illness prevalence was also calculated for each week of the season by dividing the number of players reporting an illness by the number of questionnaire responses for that week. If a player reported multiple illness episodes, these were included as separate events. Illnesses present at the start of the season were included in prevalence calculations. According to the 2020 International Olympic Committee's consensus recommendations, we grouped illness symptoms into clusters post study.[17] Sex differences in average weekly illness prevalence were compared using a prevalence rate ratio (PRR) and corresponding 95% CI. The frequency of reported illness symptoms was compared between sexes using the chi-square test, treating each athlete's weekly illness reports as independent observations, and mean time loss (number of days absence) per illness week using the Student's t-test. Data were analysed using SPSS® 25.0 (IBM SPSS Statistics 2015). The significance level was set to $p<0.05$.

138 **Results**

139 There were 471 players included in 47 teams. The mean age for the 329 male and 142 female players was 13.3 (SD 1.0) and 13.7 (SD 1.5) years, respectively. The mean number of weekly reports per player was 14.4 (SD 7.8), with 13.5 (SD 8.1) for males and 16.3 (SD 6.8) for females. In total, 61% of players (n=288, 60% males vs 64% females) reported at least one illness during the season. Of the total number of 6699 weekly reports with complete data for OSTRC question 1 (data were missing for 59 reports), 74% (76% male vs 71% female) indicated that players were able to fully participate in floorball without health problems, in 14% (n=948) the player reported an injury (and hence could not report an illness), and in 12% (n=781) of all reports the player reported an illness. Among the 781 weeks with a reported illness, the player reported full floorball participation in 196 weeks (25%; 21%

For peer review only - http://bmjopen.bmj.com/site/about/guidelines.xhtml

males vs 31% females), reduced participation in 202 weeks (26%; 26% for both males and females), and inability to participate in floorball due to illness in 383 weeks (49%; 53% males vs 43% females). The mean (SD) time loss from training and matches for all illness weeks was 2.0 (1.7) days, with no difference between sexes (male 2.0 (1.6) vs female 1.9 (1.7) days, p=0.340).

Illness prevalence during the season

The average weekly illness prevalence for the whole season was 12% (95% CI 10.8–12.3%) for all players, with a slightly higher prevalence among females (13%, 95% CI 11.6–14.3%) than males (11%, 95% CI 9.9–11.7%), PRR 1.20 (95% CI 1.05–1.37, p=0.009). Illness prevalence ranged from 5% to 25% in different teams.

The weekly illness prevalence ranged from 5% to 18% over the 26 weeks (4–23% for female vs 5%–17% for male players). Illness prevalence was highest in calendar weeks 4 to 7 (peak winter weeks in January/February) and ranged from 15% to 18% during this period (20–23% for females vs 12–17% for males) (Figure 1).

<Insert "**Figure 1**: Weekly illness prevalence for all players and by sex during the 26-week floorball season" here>

Illness symptoms

The most common self-reported illness symptoms were fever (30%), sore throat (16%) and cough (14%). Female players more often reported difficulty in breathing/tight airways and fainting, and male players more frequently reported coughing, feeling tired/feverish and headache (Table 2).

For peer review only - http://bmjopen.bmj.com/site/about/guidelines.xhtml

171 **Table 2**: Self-reported illness symptoms reported during the 26-week floorball season sorted by illness symptom cluster

Illness symptom clusters	All	Female	Male	P-value female vs male	Illness impact on participation in floorball		
					Full participation but with illness	Reduced participation due to illness	Could not participate due to illness
**Upper/lower respiratory (total)**	**387 (49.9)**	**156 (52.7)**	**231 (48.1)**	**0.215**	**133**	**104**	**150**
- Sore throat	122 (15.7)	46 (15.5)	76 (15.8)	0.913	22	42	58
- Coughing	105 (13.5)	30 (10.1)	75 (15.6)	0.030	36	23	46
- Nasal congestion/runny nose/sneezing	100 (12.9)	32 (10.8)	68 (14.2)	0.175	30	29	41
- Difficulty breathing/tight airways	60 (7.7)	48 (16.2)	12 (2.5)	<0.001	45	10	5
**Non-specific illness (total)**	**278 (35.8)**	**96 (32.5)**	**182 (37.9)**	**0.122**	**19**	**72**	**187**
- Fever	230 (29.6)	86 (29.1)	144 (30.0)	0.779	9	48	173
- Feeling tired/feverish	42 (5.4)	8 (2.7)	34 (7.1)	0.009	9	23	10
- Other pain	6 (0.8)	2 (0.7)	4 (0.8)	NC	1	1	4
**Neurological - Headache**	**32 (4.1)**	**4 (1.4)**	**28 (5.9)**	**0.002**	**12**	**10**	**10**
**Gastrointestinal (total)**	**25 (3.3)**	**8 (2.8)**	**17 (3.5)**	**0.520**	**4**	**10**	**11**
- Nausea	14 (1.8)	4 (1.4)	10 (2.1)	0.457	2	6	6
- Vomiting	6 (0.8)	2 (0.7)	4 (0.8)	NC	-	1	5
- Abdominal pain	3 (0.4)	2 (0.7)	1 (0.2)	NC	2	1	-
- Diarrhoea	2 (0.3)	-	2 (0.4)	NC	-	2	-
**Cardiovascular - Fainting**	**20 (2.6)**	**20 (6.8)**	**-**	**NC**	**17**	**1**	**2**
**Other - Unspecified**	**27 (3.5)**	**8 (2.7)**	**19 (4.0)**	**0.354**	**6**	**5**	**16**
- Swollen glands	10 (1.3)	2 (0.7)	8 (1.7)	NC	2	-	8
**Otological - Ear symptoms**	**3 (0.4)**	**2 (0.7)**	**1 (0.2)**	**NC**	**1**	**-**	**2**
**Psychological (total)**	**3 (0.4)**	**2 (0.7)**	**1 (0.2)**	**NC**	**1**	**-**	**2**
- Anxiety	2 (0.3)	1 (0.3)	1 (0.2)	NC	1	-	1
- Irritation	1 (0.1)	1 (0.3)	-	NC	-	-	1
**Ophthalmological - Eye symptoms**	**1 (0.1)**	**-**	**1 (0.2)**	**NC**	**1**	**-**	**-**
**Total**	**776* (100)**	**296 (100)**	**480 (100)**		**194**	**202**	**380**

172 Values are number of weekly reports (% of total weekly reports) with each reported symptom. *Illness symptoms missing for 5 weekly illness reports. P-
173 values for comparison of illness symptom frequency between sexes using the chi-square test (not calculated (NC) if expected value in any cell is less than 5).

For peer review only - http://bmjopen.bmj.com/site/about/guidelines.xhtml

Weekly symptom prevalence for non-specific illness symptoms (fever, feeling tired/feverish) and upper/lower respiratory symptoms (sore throat, coughing, nasal congestion/runny nose/sneezing, difficulty breathing/tight airways) were high during January and February (weeks 4–7). Respiratory symptoms also peaked in mid-October (Figure 2).

<Insert "**Figure 2**: Weekly symptom prevalence for all players during the 26-week floorball season" here>

Discussion

The main findings of this study were that: (1) 61% of youth floorball players reported at least one illness week during the season, with an average weekly illness prevalence of 12%, (2) 49% of all weekly reports with an illness indicated that the player was not able to participate in floorball (time loss), (3) illness prevalence was slightly higher among females, (4) fever (30%) and upper and lower respiratory symptoms such as sore throat (16%), cough (14%) and nasal symptoms (13%) were the most common, and (5) illness prevalence was highest in the peak winter months (late January/February), affecting 15% to 18% of the players during this period.

Illness prevalence and symptoms

The average weekly illness prevalence was 12%, which is comparable to Norwegian youth elite athletes participating in technical sports (10%),[8] but higher than for Norwegian elite youth athletes participating in team sports (8–9%),[8 18] Dutch junior tennis players (6%),[10] and Irish elite youth track and field athletes (7%).[19] The Swedish floorball season runs from September to March (autumn and winter months) when illness is more prevalent compared to summer months when athletics and tennis are played. Therefore, youth players are likely to be exposed to different viruses in school or

For peer review only - http://bmjopen.bmj.com/site/about/guidelines.xhtml

public places. Also, some established norms associated with team sports, ranging from sharing drinking bottles, towels, equipment and locker rooms, to social behaviour such as hugging, shaking hands and celebrations can enhance transmission of air- and droplet-borne pathogens like influenza.

Sports clubs can implement various behavioural, lifestyle and medical intervention strategies to ensure that sporting environments are safe spaces for sports participation, and this has become even more crucial during the current COVID-19 pandemic.[9 20 21] The education of athletes, coaches, parents and support personnel about risk-mitigation strategies for viral infections is important. This includes physical distancing to reduce the risk of transmission, cough and sneeze etiquette, wearing masks when symptomatic (coughing, sneezing) to reduce the transmission of potentially infectious respiratory droplets, regular hand hygiene, not sharing equipment such as drinking bottles, and strategies that facilitate good quality sleep and sleep hygiene practices.[9] Improved health literacy, including awareness of self-monitoring of respiratory symptoms (even if mild), could help to reduce the risk of spreading the infection to team mates.[20] Medical strategies also include implementing illness prevention guidelines, illness surveillance and respiratory symptom screening, identifying at-risk and vulnerable individuals and implementing full preventative precautions during high-risk periods.[9]

The most common symptoms were fever (30%), and upper/lower respiratory symptoms such as sore throat (16%), cough (14%) and nasal symptoms (13%). These symptoms peaked during the winter season in late January/February (weeks 4–7) in our floorball cohort, which was concurrent with a sharp increase in influenza B/Yamagata in the general population of young people in Sweden during week 3, with a peak in week 7.[22] This indicates that influenza could be one common cause of illness in our Swedish youth floorball cohort.

Our data show that one in every two reported illnesses led to complete time loss from the sport, with a mean absence of 2 days per illness week. Participating in sports during febrile illness can worsen the illness or lead to other detrimental outcomes and return to activity is only recommended

For peer review only - http://bmjopen.bmj.com/site/about/guidelines.xhtml

with the resolution of fever.[23] Therefore it is worrisome that 1 in 4 athletes who reported fever as the main symptom, and 3 of 4 athletes who reported feeling tired/feverish, also reported full or partial participation in floorball.

Comparison between sexes

We observed a slightly higher weekly illness prevalence in female youth floorball players than males (13% vs 11%), and our findings concur with the results from several different sports in Norwegian youth elite athletes.[8 18] In elite adult sports as well, female athletes appear to be more prone to illness,[9] yet the influence of sex on illness prevalence remains unclear in elite youth sports.[10-13] Overall, reported symptom clusters were similar between sexes, whilst among upper/lower respiratory symptoms more female players (16% vs 3% males) reported difficulty in breathing/tight airways, while males more commonly reported coughing (16% vs 10% females). Furthermore, approximately one in 20 female players reported fainting, yet no male players did so. The reason for this sex discrepancy is unclear.

Methodological considerations

Self-reported illness data relies on young players' memory, and consequently is subject to recall bias. The use of prospective weekly reports limits the recall period to one week. Additionally, self-reported illness reports are open to interpretation bias of the survey question, and it is possible that young people may not fully understand illness-related questions. Question responses were standardised by providing a list of symptoms to collect self-reported data as accurately as possible. Since the diagnosis was not available from self-reported illness, we followed the 2020 International Olympic Committee's methods for reporting illness data by symptoms, and symptom clusters.[17] The study was limited by a low overall survey response rate (61%), which is similar to two previous studies in Swedish and Norwegian youth elite athletes (response rates 60-66%).[8 24] Female players had a higher survey response rate than males (16 vs 14 weekly reports during the season). It is unknown whether there is a bias towards higher or lower response rates in weeks when the athlete

For peer review only - http://bmjopen.bmj.com/site/about/guidelines.xhtml

had experienced a health problem, thus risking either over- or under-inflation of the illness prevalence rate. Owing to the skip logic application in our survey, the response burden is low for those who report illness. The sample of female floorball players was smaller than that of male players and thus may be less robust. Because of the way in which the survey was structured, there is a possibility that illness data might be under-reported. Injury was the primary outcome of interest in the intervention study, and if a player reported an injury in the weekly survey they could not report a concurrent illness in the same week. Therefore, our illness prevalence numbers represent the minimum illness rates in the study cohort. If we assume the same illness prevalence during injury weeks as non-injury weeks (12%), the "true" weekly illness prevalence in our cohort would be slightly higher at 13% (95% CI 12.4–14.0%).

Conclusions

This prospective study showed that 61% of youth floorball players reported at least one illness week during the season, with an average weekly illness prevalence of 12%. Our novel findings relating to the prevalence and pattern of illness in youth floorball may help to direct prevention strategies. Athletes, coaches, parents and support personnel need to be educated about risk mitigation strategies for common infections. Improved awareness about the self-monitoring of respiratory symptoms could help to reduce the risk of spreading the infection to team mates.

Acknowledgements

The authors would like to thank Taru Tervo, PhD, School of Sport Sciences, Umeå University, and Emil Risberg, of The Swedish Floorball Federation, for input on the study plan, surveys and research questions, as well as administrative assistance. We also thank the participating clubs, coaches and players for their participation in the study. The Sport Without Injury ProgrammE (SWIPE) was

For peer review only - http://bmjopen.bmj.com/site/about/guidelines.xhtml

established at Linköping University, Linköping, Sweden, through grants from the Swedish Research Council and the Swedish National Centre for Research in Sports.

Author contributions

NKPP analysed and interpreted the data and produced the first draft of the manuscript. MW, HL, IÅ and SS provided critical comments on the manuscript. MH conceptualised the study, interpreted the data and provided critical comments on the manuscript. All authors were involved in designing the study and approved the final draft for submission.

Data sharing statement

De-identified data may be shared upon reasonable request.

Patient and public involvement

The Swedish Floorball Federation and the Floorball Competence Centre (Innebandyns Kompetenscentrum) at Umeå University were included in the planning and preparation of this study. Their involvement included input on the study plan, surveys and research questions, help with the recruitment of participants, and plans for disseminating the study results to participants and wider relevant communities. They had no influence on the analyses, interpretation of results or manuscript preparation.

Patient consent for publication

Not required.

Checklist and flow diagram for the appropriate reporting statement

STROBE SIIS checklist

For peer review only

For peer review only - http://bmjopen.bmj.com/site/about/guidelines.xhtml

Licence statement

I, the Submitting Author have the right to grant and do grant on behalf of all authors of the Work (as defined in the below author licence), an exclusive licence and/or a non-exclusive licence for contributions from authors who are: i) UK Crown employees; ii) where BMJ has agreed a CC-BY licence shall apply, and/or iii) in accordance with the terms applicable for US Federal Government officers or employees acting as part of their official duties; on a worldwide, perpetual, irrevocable, royalty-free basis to BMJ Publishing Group Ltd ("BMJ") its licensees and where the relevant Journal is co-owned by BMJ to the co-owners of the Journal, to publish the Work in BMJ Open and any other BMJ products and to exploit all rights, as set out in our licence.

The Submitting Author accepts and understands that any supply made under these terms is made by BMJ to the Submitting Author unless you are acting as an employee on behalf of your employer or a postgraduate student of an affiliated institution which is paying any applicable article publishing charge ("APC") for Open Access articles. Where the Submitting Author wishes to make the Work available on an Open Access basis (and intends to pay the relevant APC), the terms of reuse of such Open Access shall be governed by a Creative Commons licence – details of these licences and which Creative Commons licence will apply to this Work are set out in our licence referred to above.

Figure legends

Figure 1: Weekly illness prevalence for all players and by sex during the 26-week floorball season

Figure 2: Weekly symptom prevalence for all players during the 26-week floorball season

For peer review only

For peer review only - http://bmjopen.bmj.com/site/about/guidelines.xhtml

References

1. Raysmith B, Drew M. Performance success or failure is influenced by weeks lost to injury and illness in elite Australian track and field athletes: a 5-year prospective study. *J Sci Med Sport* 2016;19(10):778-83.

2. Schwellnus M. The Olympic textbook of medicine in sport. John Wiley & Sons 2009:344-65.

3. Van Tonder A, Schwellnus M, Swanevelder S, et al. A prospective cohort study of 7031 distance runners shows that 1 in 13 report systemic symptoms of an acute illness in the 8–12 day period before a race, increasing their risk of not finishing the race 1.9 times for those runners who started the race: SAFER study IV. *Br J Sports Med* 2016;50(15):939-45.

4. Timpka T, Jacobsson J, Bargoria V, et al. Preparticipation predictors for championship injury and illness: cohort study at the Beijing 2015 International Association of Athletics Federations World Championships. *Br J Sports Med* 2017;51(4):271-76.

5. Friman G, Wesslen L. Infections and exercise in high-performance athletes. *Immunol Cell Biol* 2000;78(5):510-22.

6. Tseng GS, Hsieh CY, Hsu CT, et al. Myopericarditis and exertional rhabdomyolysis following an influenza A (H3N2) infection. *BMC Infect Dis* 2013;13:283.

7. Weidner T, Sevier T. Sport, exercise, and the common cold. *J Athl Train* 1996;31(2):154-59.

8. Moseid C, Myklebust G, Fagerland M, et al. The prevalence and severity of health problems in youth elite sports: A 6-month prospective cohort study of 320 athletes. *Scand J Med Sci Sports* 2018;28(4):1412-23.

9. Schwellnus M, Soligard T, Alonso J-M, et al. How much is too much? (Part 2) International Olympic Committee consensus statement on load in sport and risk of illness. *Br J Sports Med* 2016;50(17):1043-52.

10. Pluim B, Loeffen F, Clarsen B, et al. A one-season prospective study of injuries and illness in elite junior tennis. *Scand J Med Sci Sports* 2016;26(5):564-71.

For peer review only - http://bmjopen.bmj.com/site/about/guidelines.xhtml

11. Ruedl G, Schobersberger W, Pocecco E, et al. Sport injuries and illnesses during the first Winter Youth Olympic Games 2012 in Innsbruck, Austria. *Br J Sports Med* 2012;46(15):1030-7.

12. Steffen K, Moseid C, Engebretsen L, et al. Sports injuries and illnesses in the Lillehammer 2016 Youth Olympic Winter Games. *Br J Sports Med* 2017;51(1):29-35.

13. Steffen K, Soligard T, Mountjoy M, et al. How do the new Olympic sports compare with the traditional Olympic sports? Injury and illness at the 2018 Youth Olympic Summer Games in Buenos Aires, Argentina. *Br J Sports Med* 2020;54(3):168-75.

14. Åkerlund I, Waldén M, Sonesson S, et al. Forty-five per cent lower acute injury incidence but no effect on overuse injury prevalence in youth floorball players (aged 12–17 years) who used an injury prevention exercise programme: two-armed parallel-group cluster randomised controlled trial. *Br J Sports Med* 2020;54(17):1028-35.

15. Perera N, Åkerlund I, Hägglund M. Motivation for sports participation, injury prevention expectations, injury risk perceptions and health problems in youth floorball players. *Knee Surg Sports Traumatol Arthrosc* 2019;27(11):3722-32.

16. Clarsen B, Rønsen O, Myklebust G, et al. The Oslo Sports Trauma Research Center questionnaire on health problems: a new approach to prospective monitoring of illness and injury in elite athletes. *Br J Sports Med* 2014;48(9):754-60.

17. Bahr R, Clarsen B, Derman W, et al. International Olympic Committee consensus statement: methods for recording and reporting of epidemiological data on injury and illness in sport 2020 (including STROBE Extension for Sport Injury and Illness Surveillance (STROBE-SIIS)). *Br J Sports Med* 2020;54(7):372-89.

18. Bjørndal C, Bache-Mathiesen L, Gjesdal S, et al. An examination of training load, match activities, and health problems in Norwegian youth elite handball players over one competitive season. *Front Sports Act Living* 2021;3(36)

For peer review only

For peer review only - http://bmjopen.bmj.com/site/about/guidelines.xhtml

19. Carragher P, Rankin A, Edouard P. A one-season prospective study of illnesses, acute, and overuse injuries in elite youth and junior track and field athletes. *Front Sports Act Living* 2019;1(13)

20. Hughes D, Saw R, Perera N, et al. The Australian Institute of Sport framework for rebooting sport in a COVID-19 environment. *J Sci Med Sport* 2020;23(7):639-63.

21. Schwellnus M, Janse van Rensburg C, Bayne H, et al. Team illness prevention strategy (TIPS) is associated with a 59% reduction in acute illness during the Super Rugby tournament: a control–intervention study over 7 seasons involving 126 850 player days. *Br J Sports Med* 2020;54(4):245-49.

22. Brytting M, Dahl H, Wiman Å, et al. Influenza in Sweden 2017–2018. Public Health Agency of Sweden, Stockholm, Sweden 2018.

23. Dick NA, Diehl JJ. Febrile illness in the athlete. *Sports Health* 2014;6(3):225-31.

24. von Rosen P, Heijne A, Frohm A, et al. High injury burden in elite adolescent athletes: a 52-week prospective study. *J Athl Train* 2018;53(3):262-70.

For peer review only

For peer review only - http://bmjopen.bmj.com/site/about/guidelines.xhtml

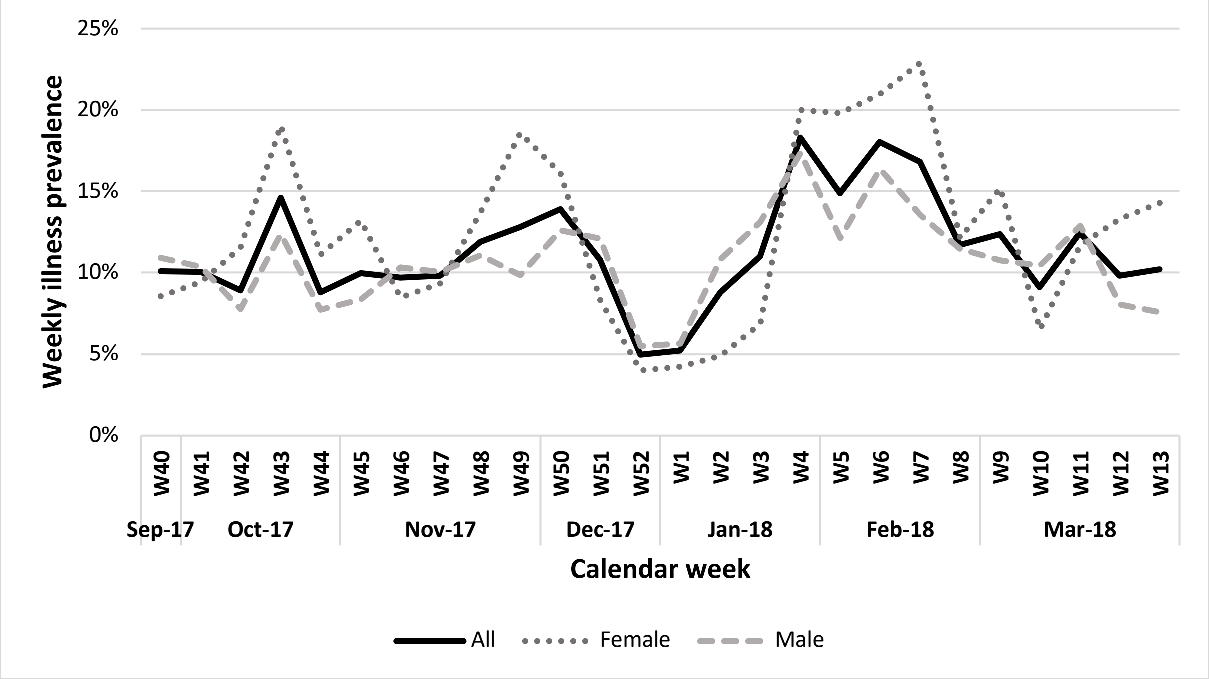

Figure 1: Weekly illness prevalence for all players and by sex during the 26-week floorball season Many teams had reduced training during weeks 52 and 1 due to Christmas and New year holidays.

For peer review only

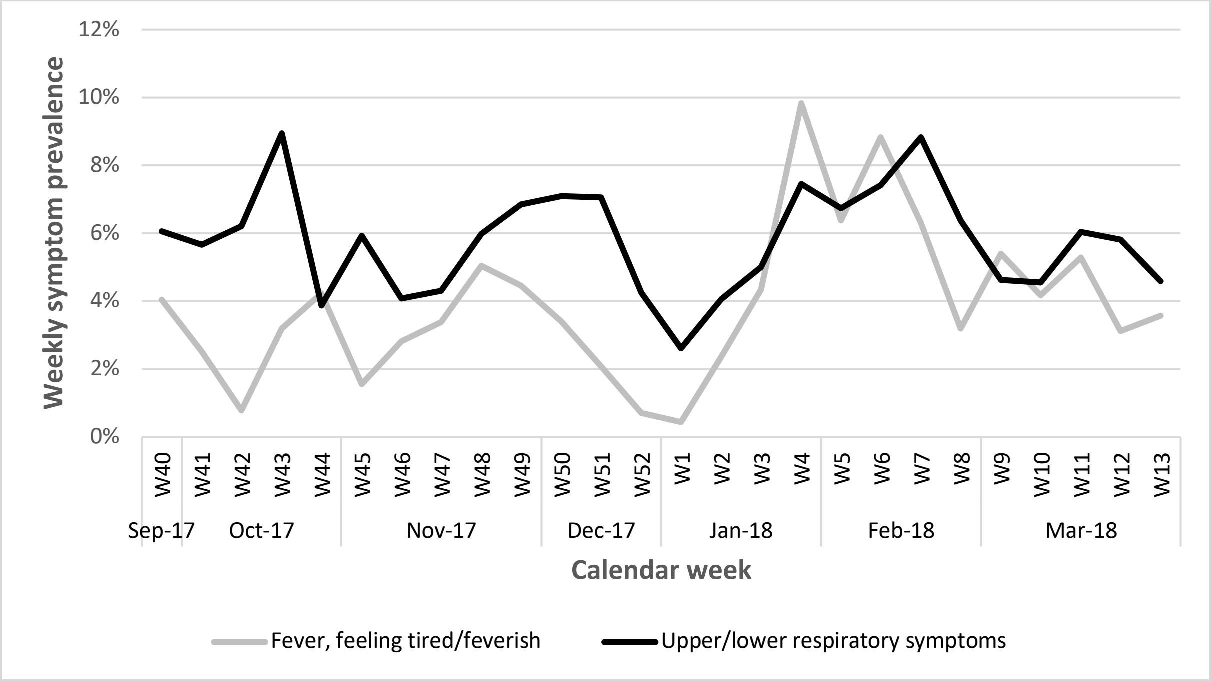

Key: Upper/lower respiratory symptoms include sore throat, coughing, nasal congestion/runny nose/sneezing, difficulty breathing/tight airways

Figure 2: Weekly symptom prevalence for all players during the 26-week floorball season

For peer review only

Appendix 3: STROBE-SIIS (Sports Injury and Illness Surveillance) Statement 1.0—Checklist of items for the reporting of observational studies on injury and illness in sports

Item	Item No	Recommendation from the STROBE Statement	STROBE-SIIS Extension +	Reported on Page #
**Title and abstract**	1	(*a*) Indicate the study's design with a commonly used term in the title or the abstract		1-2
		(*b*) Provide in the abstract an informative and balanced summary of what was done and what was found	SIIS-1.1. Include information on the sport, athlete population (sex, age, geographic region) and level of competition.<br>SIIS-1.2. Include the duration of observation (e.g. one season, one year, multiple years).	2
**Introduction**				
Background/rationale	2	Explain the scientific background and rationale for the investigation being reported		4
Objectives	3	State specific objectives, including any pre-specified hypotheses	SIIS-3.1. State whether study was registered. Identify the registration number and database used.<br>SIIS-3.2. State the specific purpose of your study (e.g. to describe the injury burden associated with Olympic level rowing)	4
**Methods**				
Study design	4	Present key elements of study design early in the paper	SIIS-4.1. Clearly specify which health problems are being observed.	5-6

For peer review only

Bahr R, *et al. Br J Sports Med* 2020; 54:372–389. doi: 10.1136/bjsports-2019-101969

Item	Item No	Recommendation from the STROBE Statement	STROBE-SIIS Extension +	
			SIIS-4.2. State explicitly which approach was used to record the health problem data, including all outcome measures or tools	5-6
			SIIS-4.3. State explicitly which coding system was used to classify the health problems (e.g. OSIICS, SMDCS, ICD, etc.)	5-7
			SIIS-4.4. Where relevant, clearly describe how athletes were categorised. Variables to consider could include the type of athlete and/or sport, the environment in which the sport occurs (e.g. type of course or playing area), the typical duration of the sport, the degree of physical contact permitted in the sport, and the equipment permitted.	5
Setting	5	Describe the setting, locations, and relevant dates, including periods of recruitment, exposure, follow-up, and data collection	SIIS-5.1. Describe the location, level of play, dates of observation and data collection methods (i.e. who, what, where).	5
			SIIS-5.2. Specify the dates of the surveillance period and how the data were handled when the study covered more than one season/calendar year.	5
			SIIS-5.3. Define whether the health problem data were collected prospectively or retrospectively.	4-5

For peer review only

Bahr R, *et al. Br J Sports Med* 2020; 54:372–389. doi: 10.1136/bjsports-2019-101969

Item	Item No	Recommendation from the STROBE Statement	STROBE-SIIS Extension +	Reported on Page #
Participants	6	(*a*) *Cohort study*—Give the eligibility criteria, and the sources and methods of selection of participants. Describe methods of follow-up<br>*Case-control study*—Give the eligibility criteria, and the sources and methods of case ascertainment and control selection. Give the rationale for the choice of cases and controls<br>*Cross-sectional study*—Give the eligibility criteria, and the sources and methods of selection of participants	SIIS-6.1. Define the population of athletes and how they were selected and recruited.	5
		(*b*) *Cohort study*—For matched studies, give matching criteria and number of exposed and unexposed<br>*Case-control study*—For matched studies, give matching criteria and the number of controls per case		
Variables	7	Clearly define all outcomes, exposures, predictors, potential confounders, and effect modifiers. Give diagnostic criteria, if applicable	SIIS-7.1. Justify why you measured your primary and secondary outcomes of interest in the specific way chosen.<br>SIIS-7.2. Describe the method for identifying your health problem outcome of interest.	6-7
Data sources/ measurement	8*	For each variable of interest, give sources of data and details of methods of assessment (measurement). Describe	SIIS-8.1. Specify who collected/reported the data for the	5

For peer review only - http://bmjopen.bmj.com/site/about/guidelines.xhtml

Item	Item No	Recommendation from the STROBE Statement	STROBE-SIIS Extension +	Reported on Page #
		comparability of assessment methods if there is more than one group	study and their qualifications (e.g. qualified doctor, data analyst, etc.).	
			SIIS-8.2. Specify who coded the data for the study and their qualifications (e.g. qualified doctor, data analyst, etc.). In many instances, this will not be the same as SIIS-8.1.	5
			SIIS-8.3. Specify the direct methods used to collect the data, and the use of physical documents or an electronic tools. If extracting information from existing sources, specify the data source.	5
			SIIS-8.4. Specify the timing of and window for data collection (e.g. day health problem occurred or following day). Specify the frequency of data collection (e.g. daily, weekly, monthly).	5
			SIIS-8.5. Report the duration of surveillance (e.g. tournament, season, whole year, playing career).	5
Bias	9	Describe any efforts to address potential sources of bias	SIIS-9.1. Clearly report any validation or reliability assessment of the data collection of tools.	
			SIIS-9.2. Formally acknowledge any potential biases in associated with the data collection method (e.g. self-	13

For peer review only

Bahr R, *et al. Br J Sports Med* 2020; 54:372–389. doi: 10.1136/bjsports-2019-101969

**Item**	**Item No**	**Recommendation from the STROBE Statement**	**STROBE-SIIS Extension** +	Reported on Page #
			report, recall bias, reporting by non-medically trained staff, etc.)	
Study size	10	Explain how the study size was arrived at		5, 10
Quantitative variables	11	Explain how quantitative variables were handled in the analyses. If applicable, describe which groupings were chosen and why	SIIS-11.1 Explain in detail how multiple injuries/illness episodes are handled both in individual athletes and across athletes/surveillance periods.	7
			SIIS-11.2. Specify how injury severity was calculated.	N/A
Statistical methods	12	(*a*) Describe all statistical methods, including those used to control for confounding	SIIS-12.1. Specify how exposure to risk has been adjusted for and specify the units (e.g. per participant, per athlete exposure, etc.).	6-7
			SIIS-12.2 Specify how relevant risk measures (incidence, prevalence, etc.) were calculated.	6-7
			SIIS-12.3. When relevant to the study aim, specify how injury burden was calculated and analysed.	N/A
		(*b*) Describe any methods used to examine subgroups and interactions		6-7
		(*c*) Explain how missing data were addressed	SIIS-12.4. For studies reporting multiple health problems, state clearly how these were handled (e.g. time to the first injury only, ignoring	7

For peer review only - http://bmjopen.bmj.com/site/about/guidelines.xhtml

Item	Item No	Recommendation from the STROBE Statement	STROBE-SIIS Extension +	Reported on Page #
			subsequent return to play and re-injuries, or modelling of all injuries).	
			SIIS-12.5. Explain how/if athletes not included at outset (e.g. those already injured) were handled in the analyses.	7
		(*d*) *Cohort study*—If applicable, explain how loss to follow-up was addressed<br>*Case-control study*—If applicable, explain how matching of cases and controls was addressed<br>*Cross-sectional study*—If applicable, describe analytical methods taking account of sampling strategy	SIIS-12.6. In longitudinal studies, it is particularly important to explain how athlete follow-up has been managed. For example, what happened if a player was transferred to another team or has been censored (for those no longer part of the study due to removal during the observation period). Censoring can occur when athletes are removed due to transfer out of the team/study, injury/illness or due to study design.	6
		(*e*) Describe any sensitivity analyses		N/A
**Results**				
Participants	13*	(a) Report numbers of individuals at each stage of study—e.g. numbers potentially eligible, examined for eligibility, confirmed eligible, included in the study, completing follow-up, and analysed	SIIS-13.1. Clearly state the number of athletes followed-up, the number (and %) of those with the health problem and the number of problems reported among them (a median number of problems per affected athlete could be useful).	7
			SIIS-13.2. For studies over multiple seasons/years, report the total	N/A

For peer review only - http://bmjopen.bmj.com/site/about/guidelines.xhtml

Item	Item No	Recommendation from the STROBE Statement	STROBE-SIIS Extension +	Reported on Page #
			numbers of health problems for each year and numbers common to each period.	
		(b) Give reasons for non-participation at each stage	SIIS-13.3. Report how athletes removed (e.g. due to transfer of teams or time-out due to injury or illness) impact upon data at key data collection/reporting points, ideally with a flow diagram	
Descriptive data	14*	(a) Give characteristics of study participants (e.g. demographic, clinical, social) and information on exposures and potential confounders	SIIS-14.1. Include detail on the level of competition being observed (e.g. by age levels, skill level, sex, etc.).	5,7
		(b) Indicate number of participants with missing data for each variable of interest		
		(c) *Cohort study*—Summarise follow-up time (e.g. average and total amount)		
Outcome data	15*	*Cohort study*—Report numbers of outcome events or summary measures over time	SIIS-15.1. In many observational studies, individuals will sustain more than one health problem over the surveillance period. Take care to ensure descriptive data representing both the number of health problems and the number of athletes affected. It is important to represent effectively both the analysis and reporting of correct units for frequency data, i.e. the % of affected	7-9

For peer review only

Bahr R, *et al. Br J Sports Med* 2020; 54:372–389. doi: 10.1136/bjsports-2019-101969

Item	Item No	Recommendation from the STROBE Statement	STROBE-SIIS Extension +	
			athletes or the % of injuries, body regions, etc.	
		Case-control study—Report numbers in each exposure category, or summary measures of exposure		
		Cross-sectional study—Report numbers of outcome events or summary measures		
Main results	16	(*a*) Give unadjusted estimates and, if applicable, confounder-adjusted estimates and their precision (e.g. 95% confidence interval). Make clear which confounders were adjusted for and why they were included	SIIS-16.1. Report exposure-adjusted incidence or prevalence measures with appropriate confidence intervals when presenting risk measures.<br>SIIS-16.2. Report details of interest, such as mode of onset	7-10
		(*b*) Report category boundaries when continuous variables were categorized		
		(*c*) If relevant, consider translating estimates of relative risk into absolute risk for a meaningful time period		
Other analyses	17	Report other analyses done—e.g. analyses of subgroups and interactions, and sensitivity analyses	SIIS-17.1 Report injury diagnosis information, including region and tissue type in tabular form.	9
**Discussion**				
Key results	18	Summarise key results with reference to study objectives		10,14
Limitations	19	Discuss limitations of the study, taking into account sources of potential bias or	SIIS-19.1. Discuss limitations in the data collection and coding procedures adopted, including in	13

For peer review only

Bahr R, *et al. Br J Sports Med* 2020; 54:372–389. doi: 10.1136/bjsports-2019-101969

Item	Item No	Recommendation from the STROBE Statement	STROBE-SIIS Extension +	Reported on Page #
		imprecision. Discuss both direction and magnitude of any potential bias	relation to any risk measures calculated.	
Interpretation	20	Give a cautious overall interpretation of results considering objectives, limitations, multiplicity of analyses, results from similar studies, and other relevant evidence		10-14
Generalisability	21	Discuss the generalisability (external validity) of the study results	SIIS-21.1. Discuss the generalizability of the athlete study population, and health problem sub-groups of interest, to broader athlete groups.	10-14
**Other information**				
Funding	22	Give the source of funding and the role of the funders for the present study and, if applicable, for the original study on which the present article is based		3
Ethics	23		SIIS-23.1. Outline how individual athlete data privacy and confidentiality considerations were addressed, in line with the Declaration of Helsinki.	5

Note: The STROBE-SIIS checklist with additional sports epidemiology annotations should be used in conjunction with the original STROBE guideline (freely available on the Web sites of PLoS Medicine at http://www.plosmedicine.org/, Annals of Internal Medicine at http://www.annals.org/, and Epidemiology at http://www.epidem.com/). Information on the STROBE Initiative is available at www.strobe-statement.org.

+For brevity, the phrase health problem is used here to encompass both injury and illness.

For peer review only

Bahr R, *et al. Br J Sports Med* 2020; 54:372–389. doi: 10.1136/bjsports-2019-101969

*Give information separately for cases and controls in case-control studies and, if applicable, for exposed and unexposed groups in cohort and cross-sectional studies.

^^ Where there is a blank cell in this column, there are no specific additional reporting requirements for sports injury and illness surveillance over what is already covered in the original STROBE checklist.

Reference

1. Orchard O, Meeuwisse W, Derman W, et al. Refinement and presentation of the Calgary Sport Medicine Diagnostic Coding System (SMDSC) and the Orchard Sport Injury & Illness Classification System (OSIICS). *Br J Sports Med* In preparation

For peer review only

Bahr R, *et al. Br J Sports Med* 2020; 54:372–389. doi: 10.1136/bjsports-2019-101969

BMJ Open

Illness prevalence and symptoms in youth floorball players – a one-season prospective cohort study involving 471 players.

Journal:	*BMJ Open*
Manuscript ID	bmjopen-2021-051902.R2
Article Type:	Original research
Date Submitted by the Author:	12-Nov-2021

Complete List of Authors:	Panagodage Perera, Nirmala; Linköping University, Department of Health, Medicine and Caring Sciences, Unit of Physiotherapy, Sport Without Injury ProgrammE (SWIPE); Australian Institute of Sport<br>Waldén, Markus; Linköping University, Department of Health, Medicine and Caring Sciences, Unit of Physiotherapy, Sport Without Injury ProgrammE (SWIPE); Hässleholm-Kristianstad Hospitals, Department of Orthopaedics<br>Lindblom, Hanna ; Linköping University, Department of Health, Medicine and Caring Sciences, Unit of Physiotherapy, Sport Without Injury ProgrammE (SWIPE)<br>Åkerlund, Ida; Linköping University, Department of Health, Medicine and Caring Sciences, Unit of Physiotherapy, Sport Without Injury ProgrammE (SWIPE)<br>Sonesson, Sofi; Linköping University, Department of Health, Medicine and Caring Sciences, Unit of Physiotherapy, Sport Without Injury ProgrammE (SWIPE)<br>Hagglund, Martin; Linköping University, Department of Health, Medicine and Caring Sciences, Unit of Physiotherapy, Sport Without Injury ProgrammE (SWIPE)

**Primary Subject Heading**:	Sports and exercise medicine
Secondary Subject Heading:	Epidemiology
Keywords:	EPIDEMIOLOGY, SPORTS MEDICINE, PUBLIC HEALTH

SCHOLARONE™
Manuscripts

I, the Submitting Author has the right to grant and does grant on behalf of all authors of the Work (as defined in the below author licence), an exclusive licence and/or a non-exclusive licence for contributions from authors who are: i) UK Crown employees; ii) where BMJ has agreed a CC-BY licence shall apply, and/or iii) in accordance with the terms applicable for US Federal Government officers or employees acting as part of their official duties; on a worldwide, perpetual, irrevocable, royalty-free basis to BMJ Publishing Group Ltd ("BMJ") its licensees and where the relevant Journal is co-owned by BMJ to the co-owners of the Journal, to publish the Work in this journal and any other BMJ products and to exploit all rights, as set out in our licence.

The Submitting Author accepts and understands that any supply made under these terms is made by BMJ to the Submitting Author unless you are acting as an employee on behalf of your employer or a postgraduate student of an affiliated institution which is paying any applicable article publishing charge ("APC") for Open Access articles. Where the Submitting Author wishes to make the Work available on an Open Access basis (and intends to pay the relevant APC), the terms of reuse of such Open Access shall be governed by a Creative Commons licence – details of these licences and which Creative Commons licence will apply to this Work are set out in our licence referred to above.

Other than as permitted in any relevant BMJ Author's Self Archiving Policies, I confirm this Work has not been accepted for publication elsewhere, is not being considered for publication elsewhere and does not duplicate material already published. I confirm all authors consent to publication of this Work and authorise the granting of this licence.

For peer review only

Illness prevalence and symptoms in youth floorball players – a one-season prospective cohort study involving 471 players.

Perera NKP,[1,2,3] Waldén M,[2,4,5] Lindblom H,[1,2] Åkerlund I,[1,2] Sonesson S,[1,2] Hägglund M[1,2]

[1] Unit of Physiotherapy, Department of Health, Medicine and Caring Sciences (HMV), Linköping University, Linköping, Sweden

[2] Sport Without Injury ProgrammE (SWIPE), Department of Health, Medicine and Caring Sciences (HMV), Linköping University, Linköping, Sweden

[3] Sport Medicine, The Australian Institute of Sport, Burce, ACT, Australia

[4] Unit of Community Medicine, Department of Health, Medicine and Caring Sciences (HMV), Linköping University, Linköping, Sweden

[5] Department of Orthopaedics, Hässleholm-Kristianstad Hospitals, Hässleholm, Sweden

Nirmala Perera perera.nk@outlook.com

Markus Waldén markus.walden@telia.com

Hanna Lindblom hanna.lindblom@liu.se

Ida Åkerlund ida.akerlund@liu.se

Sofi Sonesson sofi.sonesson@liu.se

Martin Hägglund (corresponding author) martin.hagglund@liu.se Unit of Physiotherapy, Department of Health, Medicine and Caring Sciences (HMV), Linköping University, 581 83 Linköping, Sweden. Tel +46 733347704

Keywords: Sport, Illness, Youth, Epidemiology

Word count: 2523

For peer review only - http://bmjopen.bmj.com/site/about/guidelines.xhtml

Abstract

Objective: To describe weekly illness prevalence and illness symptoms by sex in youth floorball players during one season.

Design: Prospective cohort study

Setting: Players who were registered to play community level floorball during the 2017–2018 season (26 weeks) in two provinces in southern Sweden.

Participants: 471 youth players aged 12–17 years. Mean (SD) age for 329 male players 13.3 (1.0) years and 142 female players 13.7 (1.5) years.

Primary and secondary outcome measures: Weekly self-reported illness prevalence and illness symptoms according to the 2020 International Olympic Committee's consensus recommendations.

Results: 61% of youth floorball players reported at least one illness week during the season, with an average weekly illness prevalence of 12% (95% CI 10.8–12.3%). The prevalence was slightly higher among females (13%, 95% CI 11.6–14.3%) than males (11%, 95% CI 9.9–11.7%), prevalence rate ratio 1.20 (95% CI 1.05–1.37, p=0.009). In total, 49% (53% male, 43% female) of illness reports indicated that the player could not participate in floorball (time loss), with a mean (SD) absence of 2.0 (1.7) days per illness week. Fever (30%), sore throat (16%) and cough (14%) were the most common symptoms. Female players more often reported difficulty in breathing/tight airways and fainting, and male players more often reported coughing, feeling tired/feverish and headache. Illness prevalence was highest in the peak winter months (late January/February) reaching 15–18% during this period.

Conclusions: Our novel findings of the illness prevalence and symptoms in youth floorball may help direct prevention strategies. Athletes, coaches, parents and support personnel need to be educated about risk mitigation strategies.

For peer review only

Trial registration: Clinical Trials Registration NCT03309904

Article Summary – Strengths and limitations of this study

- The first study to evaluate self-reported prevalence, seasonal variations and illness symptom clusters by sex according to the 2020 International Olympic Committee's methods for recording and reporting epidemiological data consensus recommendations.
- Illness data from a large sample of male and female recreational youth floorball players over a 26-week period.
- Self-reported illness data may be subject to recall and interpretation bias, and the actual diagnosis was not available.
- Injury was the primary outcome of interest in the intervention study; players who reported an injury in the weekly survey could not report a concurrent illness during the same week, and illness data thus represents minimum illness prevalence rates in the study cohort.

Funding statement

The Sport Without Injury ProgrammE is funded by the Swedish Research Council (2015-02414 and 2018-03135) and the Swedish Research Council for Sport Science (P2018-0167).

Competing interests statement

None declared.

For peer review only

Introduction

Acute illness presents a significant health burden to athletes, interfering with their training, reducing sport performance and causing time loss from competitions,[1-3] and may increase the risk of subsequent injuries.[4] Strenuous exercise during acute infection can increase the risk of serious medical complications, including sudden death.[2 5-7] Performance impairments from upper respiratory tract illness may also last for 2–4 days after complete recovery.[2] Infectious illness can reduce muscle strength and impair motor coordination,[7] decrease aerobic and endurance capacity[5] and alter metabolic function[7] during and/or after the infection.

In elite youth sports, illness prevalence is higher among athletes who participate in endurance sports (23%) than those who participate in technical (10%) or team (8%) sports.[8] In elite adult sports, illness is more common among female athletes and those who participate in outdoor winter sports;[9] however, this relationship is unclear in elite youth sports.[10-13] The incidence of illness among recreational youth athletes during a season or competition has not been well studied. There is a need for increased knowledge about this population, given: 1) the scarcity of available data, 2) that most youth athletes participate in sport at recreational level, 3) it may not be appropriate to extrapolate the results from adult and elite youth cohorts to this population owing to the differences in training load (intensity and volume), competition pressure, access to resources and travelling, and 4) to provide direction for illness prevention initiatives. In this context, this study aims to be the first to: a) describe the epidemiological profile of weekly illness prevalence by sex, and b) describe self-reported illness symptoms by sex among youth floorball players during a full 26-week season.

Methods

This prospective cohort study was part of a Sport Without Injury ProgrammE (SWIPE) cluster randomised controlled trial (RCT) in floorball evaluating the efficacy of the *Knee Control* injury prevention exercise programme (Clinical Trials registration NCT03309904). Representatives from the

For peer review only - http://bmjopen.bmj.com/site/about/guidelines.xhtml

Swedish Floorball Federation informed the development of the *Knee Control* injury prevention exercise programme, and coaches (end-users) were involved in the RCT. The study was conducted in line with the Declaration of Helsinki, and approved by the Swedish Ethical Review Authority (Dnr 2017/294-31). Written informed consent was collected from all participating players, and from legal guardians for players below 15 years of age. Since the 15-minute warm-up training intervention was not believed to influence illness occurrences, we included all players in both the intervention and control group arms of the RCT for analysis in this sub-study. Youth male and female community-level floorball players aged 12–17 years from two provinces in southern Sweden (Östergötland and Småland) registered to play floorball in a club during the 2017–2018 season (26 weeks) were included.[14 15] Floorball is a winter sport played indoors on a playing surface made of rubber or parquet, using a plastic stick and light plastic ball on a 40 x 20 m pitch. There are five field players and one goalkeeper on the court in each team with free substitutes.

To prospectively collect illness data, all players completed a weekly web-based survey during the season using the Oslo Sports Trauma Research Center (OSTRC) questionnaire on health problems.[16] If players answered anything other than the first option on the OSTRC questionnaire question 1, i.e. "full participation in floorball training and matches during the past week without health problems", they were asked to indicate whether their health problem was an injury/complaint or illness. Players who selected illness then answered a question about their main illness symptoms during the past week, and which day(s), if any, they had missed scheduled floorball training or matches due to illness (Table 1). Injury was the primary outcome of interest in the intervention study, and if a player reported an injury in the weekly survey they could not report a concurrent illness during the same week. Weekly surveys were administered each Sunday at 18:00 hours via a web-link sent via short messaging service (SMS) and email to the players, using the online survey software esMakerNX3 V3. If the players did not complete the survey on the same or the following day, two additional reminders were sent via SMS on the Tuesday and Thursday. All player reports were checked by a research coordinator (physiotherapist) for completeness and accuracy, and this coordinator

For peer review only - http://bmjopen.bmj.com/site/about/guidelines.xhtml

117 contacted the player in case of any suspected errors in the registration. Player illness data were included for the full season or up until censoring, e.g. due to quitting floorball, or a season-ending injury.

120 **Table 1**: Weekly survey questions used to collect illness data during the 26-week season

**Illness**
Have you had any difficulties participating in normal floorball training during the past week due to health problems (such as pain, aches, tenderness, stiffness), injury or illness?[a]<br>– Full participation without health problems, injury or illness<br>– Full participation, but with health problems, injury or illness<br>– Reduced participation due to health problems, injury or illness<br>– Could not participate due to health problems, injury or illness
Was your current health problem an injury/complaint or illness?<br>– Injury<br>– Illness
Select the option that best matches your major illness symptoms. (Enter using the drop-down list)<br>– Fever<br>– Feeling tired/feverish<br>– Swollen glands<br>– Sore throat<br>– Nasal congestion/runny nose/sneezing<br>– Coughing<br>– Difficulty breathing/tight airways<br>– Headache<br>– Nausea<br>– Vomiting<br>– Diarrhoea<br>– Constipation<br>– Fainting<br>– Skin rash/itchy skin lesions<br>– Irregular or abnormal heartbeats, chest pain or discomfort with exercise<br>– Abdominal pain<br>– Other pain<br>– Numbness<br>– Nerve tingling/pain<br>– Anxiety<br>– Depression/feeling down<br>– Irritation<br>– Eye symptoms<br>– Ear symptoms<br>– Urogenital/gynaecological symptoms<br>– Other
Which day(s) during the past week did you miss scheduled floorball training or matches due to illness?

121 [a] Question from the Oslo Sports Trauma Research Center questionnaire on health problems[16]

For peer review only - http://bmjopen.bmj.com/site/about/guidelines.xhtml

122 A total of 7503 weekly player reports were registered over the 26 weeks; 745 weekly reports were excluded because the player was absent from floorball for the whole week due to reasons other than injury or illness (e.g. holiday). The final analysis thus comprised 6758 weekly player reports. The average weekly illness prevalence for the whole season was calculated by dividing the number of weeks a player had reported illness by the total number of weekly reports. Illness prevalence was also calculated for each week of the season by dividing the number of players reporting an illness by the number of questionnaire responses for that week. If a player reported multiple illness episodes, these were included as separate events. Illnesses present at the start of the season were included in prevalence calculations. According to the 2020 International Olympic Committee's consensus recommendations, we grouped illness symptoms into clusters post study.[17] Sex differences in average weekly illness prevalence were compared using a prevalence rate ratio (PRR) and corresponding 95% CI. The frequency of reported illness symptoms was compared between sexes using the chi-square test, treating each athlete's weekly illness reports as independent observations, and mean time loss (number of days absence) per illness week using the Student's t-test. Data were analysed using SPSS® 25.0 (IBM SPSS Statistics 2015). The significance level was set to $p<0.05$.

Results

There were 471 players included in 47 teams. The mean age for the 329 male and 142 female players was 13.3 (SD 1.0) and 13.7 (SD 1.5) years, respectively. The mean number of weekly reports per player was 14.4 (SD 7.8), with 13.5 (SD 8.1) for males and 16.3 (SD 6.8) for females. In total, 61% of players (n=288, 60% males vs 64% females) reported at least one illness during the season. Of the total number of 6699 weekly reports with complete data for OSTRC question 1 (data were missing for 59 reports), 74% (76% male vs 71% female) indicated that players were able to fully participate in floorball without health problems, in 14% (n=948) the player reported an injury (and hence could not report an illness), and in 12% (n=781) of all reports the player reported an illness. Among the 781 weeks with a reported illness, the player reported full floorball participation in 196 weeks (25%; 21%

For peer review only - http://bmjopen.bmj.com/site/about/guidelines.xhtml

males vs 31% females), reduced participation in 202 weeks (26%; 26% for both males and females), and inability to participate in floorball due to illness in 383 weeks (49%; 53% males vs 43% females). The mean (SD) time loss from training and matches for all illness weeks was 2.0 (1.7) days, with no difference between sexes (male 2.0 (1.6) vs female 1.9 (1.7) days, p=0.340).

Illness prevalence during the season

The average weekly illness prevalence for the whole season was 12% (95% CI 10.8–12.3%) for all players, with a slightly higher prevalence among females (13%, 95% CI 11.6–14.3%) than males (11%, 95% CI 9.9–11.7%), PRR 1.20 (95% CI 1.05–1.37, p=0.009). Illness prevalence ranged from 5% to 25% in different teams.

The weekly illness prevalence ranged from 5% to 18% over the 26 weeks (4–23% for female vs 5%–17% for male players). Illness prevalence was highest in calendar weeks 4 to 7 (peak winter weeks in January/February) and ranged from 15% to 18% during this period (20–23% for females vs 12–17% for males) (Figure 1).

<Insert “**Figure 1**: Weekly illness prevalence for all players and by sex during the 26-week floorball season” here>

Illness symptoms

The most common self-reported illness symptoms were fever (30%), sore throat (16%) and cough (14%). Female players more often reported difficulty in breathing/tight airways and fainting, and male players more frequently reported coughing, feeling tired/feverish and headache (Table 2).

For peer review only

For peer review only - http://bmjopen.bmj.com/site/about/guidelines.xhtml

Table 2: Self-reported illness symptoms reported during the 26-week floorball season sorted by illness symptom cluster

Illness symptom clusters	All	Female	Male	P-value female vs male	Illness impact on participation in floorball		
					Full participation but with illness	Reduced participation due to illness	Could not participate due to illness
**Upper/lower respiratory (total)**	**387 (49.9)**	**156 (52.7)**	**231 (48.1)**	**0.215**	**133**	**104**	**150**
- Sore throat	122 (15.7)	46 (15.5)	76 (15.8)	0.913	22	42	58
- Coughing	105 (13.5)	30 (10.1)	75 (15.6)	0.030	36	23	46
- Nasal congestion/runny nose/sneezing	100 (12.9)	32 (10.8)	68 (14.2)	0.175	30	29	41
- Difficulty breathing/tight airways	60 (7.7)	48 (16.2)	12 (2.5)	<0.001	45	10	5
**Non-specific illness (total)**	**278 (35.8)**	**96 (32.5)**	**182 (37.9)**	**0.122**	**19**	**72**	**187**
- Fever	230 (29.6)	86 (29.1)	144 (30.0)	0.779	9	48	173
- Feeling tired/feverish	42 (5.4)	8 (2.7)	34 (7.1)	0.009	9	23	10
- Other pain	6 (0.8)	2 (0.7)	4 (0.8)	NC	1	1	4
**Neurological - Headache**	**32 (4.1)**	**4 (1.4)**	**28 (5.9)**	**0.002**	**12**	**10**	**10**
**Gastrointestinal (total)**	**25 (3.3)**	**8 (2.8)**	**17 (3.5)**	**0.520**	**4**	**10**	**11**
- Nausea	14 (1.8)	4 (1.4)	10 (2.1)	0.457	2	6	6
- Vomiting	6 (0.8)	2 (0.7)	4 (0.8)	NC	-	1	5
- Abdominal pain	3 (0.4)	2 (0.7)	1 (0.2)	NC	2	1	-
- Diarrhoea	2 (0.3)	-	2 (0.4)	NC	-	2	-
**Cardiovascular - Fainting**	**20 (2.6)**	**20 (6.8)**	**-**	**NC**	**17**	**1**	**2**
**Other - Unspecified**	**27 (3.5)**	**8 (2.7)**	**19 (4.0)**	**0.354**	**6**	**5**	**16**
- Swollen glands	10 (1.3)	2 (0.7)	8 (1.7)	NC	2	-	8
**Otological - Ear symptoms**	**3 (0.4)**	**2 (0.7)**	**1 (0.2)**	**NC**	**1**	**-**	**2**
**Psychological (total)**	**3 (0.4)**	**2 (0.7)**	**1 (0.2)**	**NC**	**1**	**-**	**2**
- Anxiety	2 (0.3)	1 (0.3)	1 (0.2)	NC	1	-	1
- Irritation	1 (0.1)	1 (0.3)	-	NC	-	-	1
**Ophthalmological - Eye symptoms**	**1 (0.1)**	**-**	**1 (0.2)**	**NC**	**1**	**-**	**-**
**Total**	**776* (100)**	**296 (100)**	**480 (100)**		**194**	**202**	**380**

Values are number of weekly reports (% of total weekly reports) with each reported symptom. *Illness symptoms missing for 5 weekly illness reports. P-values for comparison of illness symptom frequency between sexes using the chi-square test (not calculated (NC) if expected value in any cell is less than 5).

For peer review only

For peer review only - http://bmjopen.bmj.com/site/about/guidelines.xhtml

Weekly symptom prevalence for non-specific illness symptoms (fever, feeling tired/feverish) and upper/lower respiratory symptoms (sore throat, coughing, nasal congestion/runny nose/sneezing, difficulty breathing/tight airways) were high during January and February (weeks 4–7). Respiratory symptoms also peaked in mid-October (Figure 2).

<Insert “**Figure 2**: Weekly symptom prevalence for all players during the 26-week floorball season” here>

For peer review only

Discussion

The main findings of this study were that: (1) 61% of youth floorball players reported at least one illness week during the season, with an average weekly illness prevalence of 12%, (2) 49% of all weekly reports with an illness indicated that the player was not able to participate in floorball (time loss), (3) illness prevalence was slightly higher among females, (4) fever (30%) and upper and lower respiratory symptoms such as sore throat (16%), cough (14%) and nasal symptoms (13%) were the most common, and (5) illness prevalence was highest in the peak winter months (late January/February), affecting 15% to 18% of the players during this period.

Illness prevalence and symptoms

The average weekly illness prevalence was 12%, which is comparable to Norwegian youth elite athletes participating in technical sports (10%),[8] but higher than for Norwegian elite youth athletes participating in team sports (8–9%),[8 18] Dutch junior tennis players (6%),[10] and Irish elite youth track and field athletes (7%).[19] The Swedish floorball season runs from September to March (autumn and winter months) when illness is more prevalent compared to summer months when athletics and tennis are played. Therefore, youth players are likely to be exposed to different viruses in school or

For peer review only - http://bmjopen.bmj.com/site/about/guidelines.xhtml

public places. Also, some established norms associated with team sports, ranging from sharing drinking bottles, towels, equipment and locker rooms, to social behaviour such as hugging, shaking hands and celebrations can enhance transmission of air- and droplet-borne pathogens like influenza.

Sports clubs can implement various behavioural, lifestyle and medical intervention strategies to ensure that sporting environments are safe spaces for sports participation, and this has become even more crucial during the current COVID-19 pandemic.[9 20 21] The education of athletes, coaches, parents and support personnel about risk-mitigation strategies for viral infections is important. This includes physical distancing to reduce the risk of transmission, cough and sneeze etiquette, wearing masks when symptomatic (coughing, sneezing) to reduce the transmission of potentially infectious respiratory droplets, regular hand hygiene, not sharing equipment such as drinking bottles, and strategies that facilitate good quality sleep and sleep hygiene practices.[9] Improved health literacy, including awareness of self-monitoring of respiratory symptoms (even if mild), could help to reduce the risk of spreading the infection to team mates.[20] Medical strategies also include implementing illness prevention guidelines, illness surveillance and respiratory symptom screening, identifying at-risk and vulnerable individuals and implementing full preventative precautions during high-risk periods.[9]

The most common symptoms were fever (30%), and upper/lower respiratory symptoms such as sore throat (16%), cough (14%) and nasal symptoms (13%). These symptoms peaked during the winter season in late January/February (weeks 4–7) in our floorball cohort, which was concurrent with a sharp increase in influenza B/Yamagata in the general population of young people in Sweden during week 3, with a peak in week 7.[22] This indicates that influenza could be one common cause of illness in our Swedish youth floorball cohort.

Our data show that one in every two reported illnesses led to complete time loss from the sport, with a mean absence of 2 days per illness week. Participating in sports during febrile illness can worsen the illness or lead to other detrimental outcomes and return to activity is only recommended

For peer review only - http://bmjopen.bmj.com/site/about/guidelines.xhtml

with the resolution of fever.[23] Therefore it is worrisome that 1 in 4 athletes who reported fever as the main symptom, and 3 of 4 athletes who reported feeling tired/feverish, also reported full or partial participation in floorball.

Comparison between sexes

We observed a slightly higher weekly illness prevalence in female youth floorball players than males (13% vs 11%), and our findings concur with the results from several different sports in Norwegian youth elite athletes.[8 18] In elite adult sports as well, female athletes appear to be more prone to illness,[9] yet the influence of sex on illness prevalence remains unclear in elite youth sports.[10-13] Overall, reported symptom clusters were similar between sexes, whilst among upper/lower respiratory symptoms more female players (16% vs 3% males) reported difficulty in breathing/tight airways, while males more commonly reported coughing (16% vs 10% females). Furthermore, approximately one in 20 female players reported fainting, yet no male players did so. The reason for this sex discrepancy is unclear.

Methodological considerations

Self-reported illness data relies on young players' memory, and consequently is subject to recall bias. The use of prospective weekly reports limits the recall period to one week. Additionally, self-reported illness reports are open to interpretation bias of the survey question, and it is possible that young people may not fully understand illness-related questions. Question responses were standardised by providing a list of symptoms to collect self-reported data as accurately as possible. Since the diagnosis was not available from self-reported illness, we followed the 2020 International Olympic Committee's methods for reporting illness data by symptoms, and symptom clusters.[17] The study was limited by a low overall survey response rate (61%), which is similar to two previous studies in Swedish and Norwegian youth elite athletes (response rates 60-66%).[8 24] Female players had a higher survey response rate than males (16 vs 14 weekly reports during the season). Still, our sample of female floorball players was smaller than that of male players and data may thus be less

For peer review only - http://bmjopen.bmj.com/site/about/guidelines.xhtml

robust. It is unknown whether there is a bias towards higher or lower response rates in weeks when the athlete had experienced a health problem, thus risking either over- or under-inflation of the illness prevalence rate. Because of the way in which the survey was structured, there is a possibility that illness data might be under-reported. Injury was the primary outcome of interest in the intervention study, and if a player reported an injury in the weekly survey they could not report a concurrent illness in the same week. Therefore, our illness prevalence numbers represent the minimum illness rates in the study cohort. If we assume the same illness prevalence during injury weeks as non-injury weeks (12%), the “true” weekly illness prevalence in our cohort would be slightly higher at 13% (95% CI 12.4–14.0%).

Conclusions

This prospective study showed that 61% of youth floorball players reported at least one illness week during the season, with an average weekly illness prevalence of 12%. Our novel findings relating to the prevalence and pattern of illness in youth floorball may help to direct prevention strategies. Athletes, coaches, parents and support personnel need to be educated about risk mitigation strategies for common infections. Improved awareness about the self-monitoring of respiratory symptoms could help to reduce the risk of spreading the infection to team mates.

Acknowledgements

The authors would like to thank Taru Tervo, PhD, School of Sport Sciences, Umeå University, and Emil Risberg, of The Swedish Floorball Federation, for input on the study plan, surveys and research questions, as well as administrative assistance. We also thank the participating clubs, coaches and players for their participation in the study. The Sport Without Injury ProgrammE (SWIPE) was

For peer review only

established at Linköping University, Linköping, Sweden, through grants from the Swedish Research Council and the Swedish National Centre for Research in Sports.

Author contributions

NKPP analysed and interpreted the data and produced the first draft of the manuscript. MW, HL, IÅ and SS provided critical comments on the manuscript. MH conceptualised the study, interpreted the data and provided critical comments on the manuscript. All authors were involved in designing the study and approved the final draft for submission.

Data sharing statement

De-identified data may be shared upon reasonable request.

Patient and public involvement

The Swedish Floorball Federation and the Floorball Competence Centre (Innebandyns Kompetenscentrum) at Umeå University were included in the planning and preparation of this study. Their involvement included input on the study plan, surveys and research questions, help with the recruitment of participants, and plans for disseminating the study results to participants and wider relevant communities. They had no influence on the analyses, interpretation of results or manuscript preparation.

Patient consent for publication

Not required.

Checklist and flow diagram for the appropriate reporting statement

STROBE SIIS checklist

For peer review only - http://bmjopen.bmj.com/site/about/guidelines.xhtml

Licence statement

I, the Submitting Author have the right to grant and do grant on behalf of all authors of the Work (as defined in the below author licence), an exclusive licence and/or a non-exclusive licence for contributions from authors who are: i) UK Crown employees; ii) where BMJ has agreed a CC-BY licence shall apply, and/or iii) in accordance with the terms applicable for US Federal Government officers or employees acting as part of their official duties; on a worldwide, perpetual, irrevocable, royalty-free basis to BMJ Publishing Group Ltd ("BMJ") its licensees and where the relevant Journal is co-owned by BMJ to the co-owners of the Journal, to publish the Work in BMJ Open and any other BMJ products and to exploit all rights, as set out in our licence.

The Submitting Author accepts and understands that any supply made under these terms is made by BMJ to the Submitting Author unless you are acting as an employee on behalf of your employer or a postgraduate student of an affiliated institution which is paying any applicable article publishing charge ("APC") for Open Access articles. Where the Submitting Author wishes to make the Work available on an Open Access basis (and intends to pay the relevant APC), the terms of reuse of such Open Access shall be governed by a Creative Commons licence – details of these licences and which Creative Commons licence will apply to this Work are set out in our licence referred to above.

Figure legends

Figure 1: Weekly illness prevalence for all players and by sex during the 26-week floorball season

Figure 2: Weekly symptom prevalence for all players during the 26-week floorball season

For peer review only

For peer review only - http://bmjopen.bmj.com/site/about/guidelines.xhtml

References

1. Raysmith B, Drew M. Performance success or failure is influenced by weeks lost to injury and illness in elite Australian track and field athletes: a 5-year prospective study. *J Sci Med Sport* 2016;19(10):778-83.
2. Schwellnus M. The Olympic textbook of medicine in sport. John Wiley & Sons 2009:344-65.
3. Van Tonder A, Schwellnus M, Swanevelder S, et al. A prospective cohort study of 7031 distance runners shows that 1 in 13 report systemic symptoms of an acute illness in the 8–12 day period before a race, increasing their risk of not finishing the race 1.9 times for those runners who started the race: SAFER study IV. *Br J Sports Med* 2016;50(15):939-45.
4. Timpka T, Jacobsson J, Bargoria V, et al. Preparticipation predictors for championship injury and illness: cohort study at the Beijing 2015 International Association of Athletics Federations World Championships. *Br J Sports Med* 2017;51(4):271-76.
5. Friman G, Wesslen L. Infections and exercise in high-performance athletes. *Immunol Cell Biol* 2000;78(5):510-22.
6. Tseng GS, Hsieh CY, Hsu CT, et al. Myopericarditis and exertional rhabdomyolysis following an influenza A (H3N2) infection. *BMC Infect Dis* 2013;13:283.
7. Weidner T, Sevier T. Sport, exercise, and the common cold. *J Athl Train* 1996;31(2):154-59.
8. Moseid C, Myklebust G, Fagerland M, et al. The prevalence and severity of health problems in youth elite sports: A 6-month prospective cohort study of 320 athletes. *Scand J Med Sci Sports* 2018;28(4):1412-23.
9. Schwellnus M, Soligard T, Alonso J-M, et al. How much is too much? (Part 2) International Olympic Committee consensus statement on load in sport and risk of illness. *Br J Sports Med* 2016;50(17):1043-52.
10. Pluim B, Loeffen F, Clarsen B, et al. A one-season prospective study of injuries and illness in elite junior tennis. *Scand J Med Sci Sports* 2016;26(5):564-71.

For peer review only - http://bmjopen.bmj.com/site/about/guidelines.xhtml

11. Ruedl G, Schobersberger W, Pocecco E, et al. Sport injuries and illnesses during the first Winter Youth Olympic Games 2012 in Innsbruck, Austria. *Br J Sports Med* 2012;46(15):1030-7.

12. Steffen K, Moseid C, Engebretsen L, et al. Sports injuries and illnesses in the Lillehammer 2016 Youth Olympic Winter Games. *Br J Sports Med* 2017;51(1):29-35.

13. Steffen K, Soligard T, Mountjoy M, et al. How do the new Olympic sports compare with the traditional Olympic sports? Injury and illness at the 2018 Youth Olympic Summer Games in Buenos Aires, Argentina. *Br J Sports Med* 2020;54(3):168-75.

14. Åkerlund I, Waldén M, Sonesson S, et al. Forty-five per cent lower acute injury incidence but no effect on overuse injury prevalence in youth floorball players (aged 12–17 years) who used an injury prevention exercise programme: two-armed parallel-group cluster randomised controlled trial. *Br J Sports Med* 2020;54(17):1028-35.

15. Perera N, Åkerlund I, Hägglund M. Motivation for sports participation, injury prevention expectations, injury risk perceptions and health problems in youth floorball players. *Knee Surg Sports Traumatol Arthrosc* 2019;27(11):3722-32.

16. Clarsen B, Rønsen O, Myklebust G, et al. The Oslo Sports Trauma Research Center questionnaire on health problems: a new approach to prospective monitoring of illness and injury in elite athletes. *Br J Sports Med* 2014;48(9):754-60.

17. Bahr R, Clarsen B, Derman W, et al. International Olympic Committee consensus statement: methods for recording and reporting of epidemiological data on injury and illness in sport 2020 (including STROBE Extension for Sport Injury and Illness Surveillance (STROBE-SIIS)). *Br J Sports Med* 2020;54(7):372-89.

18. Bjørndal C, Bache-Mathiesen L, Gjesdal S, et al. An examination of training load, match activities, and health problems in Norwegian youth elite handball players over one competitive season. *Front Sports Act Living* 2021;3(36)

For peer review only

358 19. Carragher P, Rankin A, Edouard P. A one-season prospective study of illnesses, acute, and overuse injuries in elite youth and junior track and field athletes. *Front Sports Act Living* 2019;1(13)

361 20. Hughes D, Saw R, Perera N, et al. The Australian Institute of Sport framework for rebooting sport in a COVID-19 environment. *J Sci Med Sport* 2020;23(7):639-63.

363 21. Schwellnus M, Janse van Rensburg C, Bayne H, et al. Team illness prevention strategy (TIPS) is associated with a 59% reduction in acute illness during the Super Rugby tournament: a control–intervention study over 7 seasons involving 126 850 player days. *Br J Sports Med* 2020;54(4):245-49.

367 22. Brytting M, Dahl H, Wiman Å, et al. Influenza in Sweden 2017–2018. Public Health Agency of Sweden, Stockholm, Sweden 2018.

369 23. Dick NA, Diehl JJ. Febrile illness in the athlete. *Sports Health* 2014;6(3):225-31.

370 24. von Rosen P, Heijne A, Frohm A, et al. High injury burden in elite adolescent athletes: a 52-week prospective study. *J Athl Train* 2018;53(3):262-70.

For peer review only

For peer review only - http://bmjopen.bmj.com/site/about/guidelines.xhtml

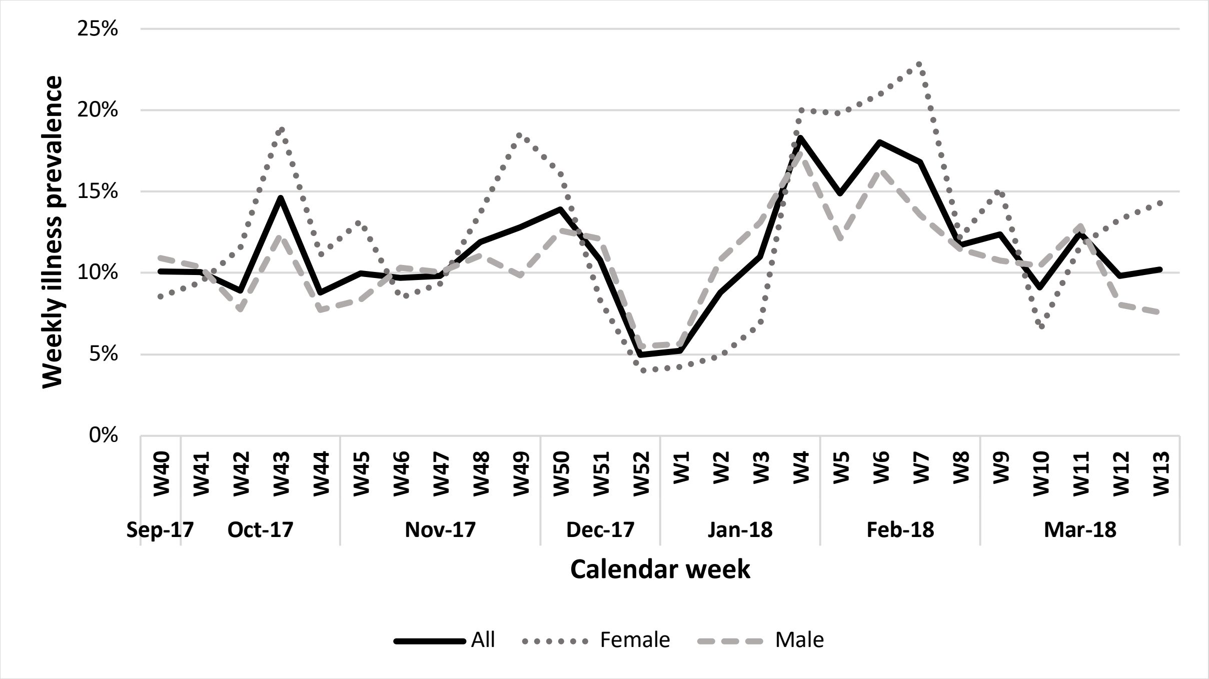

Figure 1: Weekly illness prevalence for all players and by sex during the 26-week floorball season Many teams had reduced training during weeks 52 and 1 due to Christmas and New year holidays.

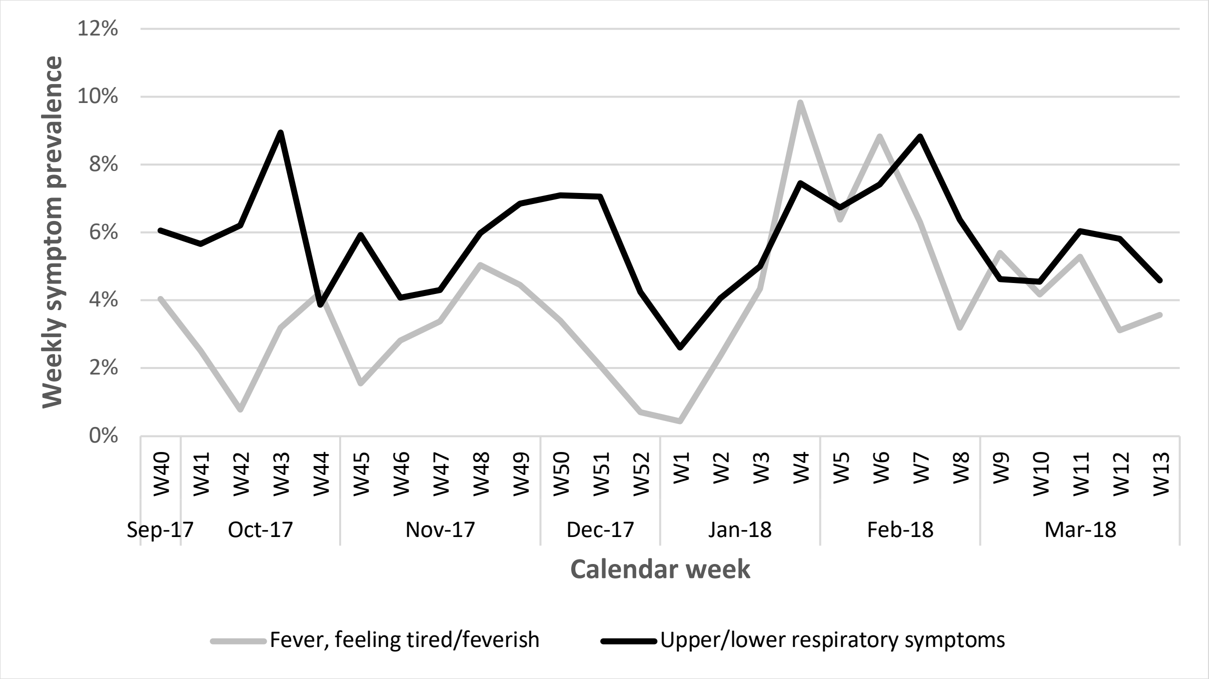

Key: Upper/lower respiratory symptoms include sore throat, coughing, nasal congestion/runny nose/sneezing, difficulty breathing/tight airways

Figure 2: Weekly symptom prevalence for all players during the 26-week floorball season

For peer review only

Appendix 3: STROBE-SIIS (Sports Injury and Illness Surveillance) Statement 1.0—Checklist of items for the reporting of observational studies on injury and illness in sports

Item	Item No	Recommendation from the STROBE Statement	STROBE-SIIS Extension +	Reported on Page #
**Title and abstract**	1	(*a*) Indicate the study's design with a commonly used term in the title or the abstract		1-2
		(*b*) Provide in the abstract an informative and balanced summary of what was done and what was found	SIIS-1.1. Include information on the sport, athlete population (sex, age, geographic region) and level of competition.<br>SIIS-1.2. Include the duration of observation (e.g. one season, one year, multiple years).	2
**Introduction**				
Background/rationale	2	Explain the scientific background and rationale for the investigation being reported		4
Objectives	3	State specific objectives, including any pre-specified hypotheses	SIIS-3.1. State whether study was registered. Identify the registration number and database used.<br>SIIS-3.2. State the specific purpose of your study (e.g. to describe the injury burden associated with Olympic level rowing)	4
**Methods**				
Study design	4	Present key elements of study design early in the paper	SIIS-4.1. Clearly specify which health problems are being observed.	5-6

For peer review only

Bahr R, *et al. Br J Sports Med* 2020; 54:372–389. doi: 10.1136/bjsports-2019-101969

Item	Item No	Recommendation from the STROBE Statement	STROBE-SIIS Extension +	
			SIIS-4.2. State explicitly which approach was used to record the health problem data, including all outcome measures or tools	5-6
			SIIS-4.3. State explicitly which coding system was used to classify the health problems (e.g. OSIICS, SMDCS, ICD, etc.)	5-7
			SIIS-4.4. Where relevant, clearly describe how athletes were categorised. Variables to consider could include the type of athlete and/or sport, the environment in which the sport occurs (e.g. type of course or playing area), the typical duration of the sport, the degree of physical contact permitted in the sport, and the equipment permitted.	5
Setting	5	Describe the setting, locations, and relevant dates, including periods of recruitment, exposure, follow-up, and data collection	SIIS-5.1. Describe the location, level of play, dates of observation and data collection methods (i.e. who, what, where).	5
			SIIS-5.2. Specify the dates of the surveillance period and how the data were handled when the study covered more than one season/calendar year.	5
			SIIS-5.3. Define whether the health problem data were collected prospectively or retrospectively.	4-5

For peer review only

Bahr R, *et al. Br J Sports Med* 2020; 54:372–389. doi: 10.1136/bjsports-2019-101969

Item	Item No	Recommendation from the STROBE Statement	STROBE-SIIS Extension +	Reported on Page #
Participants	6	(*a*) *Cohort study*—Give the eligibility criteria, and the sources and methods of selection of participants. Describe methods of follow-up<br>*Case-control study*—Give the eligibility criteria, and the sources and methods of case ascertainment and control selection. Give the rationale for the choice of cases and controls<br>*Cross-sectional study*—Give the eligibility criteria, and the sources and methods of selection of participants	SIIS-6.1. Define the population of athletes and how they were selected and recruited.	5
		(*b*) *Cohort study*—For matched studies, give matching criteria and number of exposed and unexposed<br>*Case-control study*—For matched studies, give matching criteria and the number of controls per case		
Variables	7	Clearly define all outcomes, exposures, predictors, potential confounders, and effect modifiers. Give diagnostic criteria, if applicable	SIIS-7.1. Justify why you measured your primary and secondary outcomes of interest in the specific way chosen.<br>SIIS-7.2. Describe the method for identifying your health problem outcome of interest.	6-7
Data sources/ measurement	8*	For each variable of interest, give sources of data and details of methods of assessment (measurement). Describe	SIIS-8.1. Specify who collected/reported the data for the	5

For peer review only

Bahr R, *et al. Br J Sports Med* 2020; 54:372–389. doi: 10.1136/bjsports-2019-101969

Item	Item No	Recommendation from the STROBE Statement	STROBE-SIIS Extension +	Reported on Page #
		comparability of assessment methods if there is more than one group	study and their qualifications (e.g. qualified doctor, data analyst, etc.).	
			SIIS-8.2. Specify who coded the data for the study and their qualifications (e.g. qualified doctor, data analyst, etc.). In many instances, this will not be the same as SIIS-8.1.	5
			SIIS-8.3. Specify the direct methods used to collect the data, and the use of physical documents or an electronic tools. If extracting information from existing sources, specify the data source.	5
			SIIS-8.4. Specify the timing of and window for data collection (e.g. day health problem occurred or following day). Specify the frequency of data collection (e.g. daily, weekly, monthly).	5
			SIIS-8.5. Report the duration of surveillance (e.g. tournament, season, whole year, playing career).	5
Bias	9	Describe any efforts to address potential sources of bias	SIIS-9.1. Clearly report any validation or reliability assessment of the data collection of tools.	
			SIIS-9.2. Formally acknowledge any potential biases in associated with the data collection method (e.g. self-	13

For peer review only - http://bmjopen.bmj.com/site/about/guidelines.xhtml

Item	Item No	Recommendation from the STROBE Statement	STROBE-SIIS Extension +	Reported on Page #
			report, recall bias, reporting by non-medically trained staff, etc.)	
Study size	10	Explain how the study size was arrived at		5, 10
Quantitative variables	11	Explain how quantitative variables were handled in the analyses. If applicable, describe which groupings were chosen and why	SIIS-11.1 Explain in detail how multiple injuries/illness episodes are handled both in individual athletes and across athletes/surveillance periods.	7
			SIIS-11.2. Specify how injury severity was calculated.	N/A
Statistical methods	12	(*a*) Describe all statistical methods, including those used to control for confounding	SIIS-12.1. Specify how exposure to risk has been adjusted for and specify the units (e.g. per participant, per athlete exposure, etc.).	6-7
			SIIS-12.2 Specify how relevant risk measures (incidence, prevalence, etc.) were calculated.	6-7
			SIIS-12.3. When relevant to the study aim, specify how injury burden was calculated and analysed.	N/A
		(*b*) Describe any methods used to examine subgroups and interactions		6-7
		(*c*) Explain how missing data were addressed	SIIS-12.4. For studies reporting multiple health problems, state clearly how these were handled (e.g. time to the first injury only, ignoring	7

For peer review only

For peer review only - http://bmjopen.bmj.com/site/about/guidelines.xhtml

Item	Item No	Recommendation from the STROBE Statement	STROBE-SIIS Extension +	Reported on Page #
			subsequent return to play and re-injuries, or modelling of all injuries).	
			SIIS-12.5. Explain how/if athletes not included at outset (e.g. those already injured) were handled in the analyses.	7
		(*d*) *Cohort study*—If applicable, explain how loss to follow-up was addressed<br>*Case-control study*—If applicable, explain how matching of cases and controls was addressed<br>*Cross-sectional study*—If applicable, describe analytical methods taking account of sampling strategy	SIIS-12.6. In longitudinal studies, it is particularly important to explain how athlete follow-up has been managed. For example, what happened if a player was transferred to another team or has been censored (for those no longer part of the study due to removal during the observation period). Censoring can occur when athletes are removed due to transfer out of the team/study, injury/illness or due to study design.	6
		(*e*) Describe any sensitivity analyses		N/A
**Results**				
Participants	13*	(a) Report numbers of individuals at each stage of study—e.g. numbers potentially eligible, examined for eligibility, confirmed eligible, included in the study, completing follow-up, and analysed	SIIS-13.1. Clearly state the number of athletes followed-up, the number (and %) of those with the health problem and the number of problems reported among them (a median number of problems per affected athlete could be useful).	7
			SIIS-13.2. For studies over multiple seasons/years, report the total	N/A

For peer review only

Bahr R, *et al. Br J Sports Med* 2020; 54:372–389. doi: 10.1136/bjsports-2019-101969

Item	Item No	Recommendation from the STROBE Statement	STROBE-SIIS Extension +	Reported on Page #
			numbers of health problems for each year and numbers common to each period.	
		(b) Give reasons for non-participation at each stage	SIIS-13.3. Report how athletes removed (e.g. due to transfer of teams or time-out due to injury or illness) impact upon data at key data collection/reporting points, ideally with a flow diagram	
Descriptive data	14*	(a) Give characteristics of study participants (e.g. demographic, clinical, social) and information on exposures and potential confounders	SIIS-14.1. Include detail on the level of competition being observed (e.g. by age levels, skill level, sex, etc.).	5,7
		(b) Indicate number of participants with missing data for each variable of interest		
		(c) *Cohort study*—Summarise follow-up time (e.g. average and total amount)		
Outcome data	15*	*Cohort study*—Report numbers of outcome events or summary measures over time	SIIS-15.1. In many observational studies, individuals will sustain more than one health problem over the surveillance period. Take care to ensure descriptive data representing both the number of health problems and the number of athletes affected. It is important to represent effectively both the analysis and reporting of correct units for frequency data, i.e. the % of affected	7-9

For peer review only

Bahr R, *et al. Br J Sports Med* 2020; 54:372–389. doi: 10.1136/bjsports-2019-101969

Item	Item No	Recommendation from the STROBE Statement	STROBE-SIIS Extension +	
			athletes or the % of injuries, body regions, etc.	
		Case-control study—Report numbers in each exposure category, or summary measures of exposure		
		Cross-sectional study—Report numbers of outcome events or summary measures		
Main results	16	(*a*) Give unadjusted estimates and, if applicable, confounder-adjusted estimates and their precision (e.g. 95% confidence interval). Make clear which confounders were adjusted for and why they were included	SIIS-16.1. Report exposure-adjusted incidence or prevalence measures with appropriate confidence intervals when presenting risk measures.<br>SIIS-16.2. Report details of interest, such as mode of onset	7-10
		(*b*) Report category boundaries when continuous variables were categorized		
		(*c*) If relevant, consider translating estimates of relative risk into absolute risk for a meaningful time period		
Other analyses	17	Report other analyses done—e.g. analyses of subgroups and interactions, and sensitivity analyses	SIIS-17.1 Report injury diagnosis information, including region and tissue type in tabular form.	9
**Discussion**				
Key results	18	Summarise key results with reference to study objectives		10,14
Limitations	19	Discuss limitations of the study, taking into account sources of potential bias or	SIIS-19.1. Discuss limitations in the data collection and coding procedures adopted, including in	13

For peer review only - http://bmjopen.bmj.com/site/about/guidelines.xhtml

Item	Item No	Recommendation from the STROBE Statement	STROBE-SIIS Extension +	Reported on Page #
		imprecision. Discuss both direction and magnitude of any potential bias	relation to any risk measures calculated.	
Interpretation	20	Give a cautious overall interpretation of results considering objectives, limitations, multiplicity of analyses, results from similar studies, and other relevant evidence		10-14
Generalisability	21	Discuss the generalisability (external validity) of the study results	SIIS-21.1. Discuss the generalizability of the athlete study population, and health problem sub-groups of interest, to broader athlete groups.	10-14
**Other information**				
Funding	22	Give the source of funding and the role of the funders for the present study and, if applicable, for the original study on which the present article is based		3
Ethics	23		SIIS-23.1. Outline how individual athlete data privacy and confidentiality considerations were addressed, in line with the Declaration of Helsinki.	5

Note: The STROBE-SIIS checklist with additional sports epidemiology annotations should be used in conjunction with the original STROBE guideline (freely available on the Web sites of PLoS Medicine at http://www.plosmedicine.org/, Annals of Internal Medicine at http://www.annals.org/, and Epidemiology at http://www.epidem.com/). Information on the STROBE Initiative is available at www.strobe-statement.org.

+For brevity, the phrase health problem is used here to encompass both injury and illness.

For peer review only

Bahr R, *et al. Br J Sports Med* 2020; 54:372–389. doi: 10.1136/bjsports-2019-101969

*Give information separately for cases and controls in case-control studies and, if applicable, for exposed and unexposed groups in cohort and cross-sectional studies.

^^ Where there is a blank cell in this column, there are no specific additional reporting requirements for sports injury and illness surveillance over what is already covered in the original STROBE checklist.

Reference

1. Orchard O, Meeuwisse W, Derman W, et al. Refinement and presentation of the Calgary Sport Medicine Diagnostic Coding System (SMDSC) and the Orchard Sport Injury & Illness Classification System (OSIICS). *Br J Sports Med* In preparation

For peer review only

For peer review only - http://bmjopen.bmj.com/site/about/guidelines.xhtml
